# Ex Vivo Evaluation of Poly(Solketal Acrylate) Nanoparticles for Intravitreal Drug Delivery to the Posterior Eye Segment

**DOI:** 10.1002/marc.202500788

**Published:** 2025-12-12

**Authors:** Yasaman Pourdakheli Hamedani, José Hurst, Malte Ritter, Philipp Weingarten, Julia Skokowa, Sven Schnichels, Friederike Adams

**Affiliations:** ^1^ Section for Translational Research in Ophthalmology Center for Ophthalmology University Eye Hospital Tübingen Tübingen Germany; ^2^ Department of Oncology, Hematology, Clinical Immunology and Rheumatology University Hospital Tuebingen Tuebingen Germany; ^3^ Chair of Macromolecular Chemistry TUM School of Natural Sciences Technical University of Munich Garching bei München Germany; ^4^ Chair of Macromolecular Materials and Fiber Chemistry Institute of Polymer Chemistry University of Stuttgart Stuttgart Germany

**Keywords:** ophthalmic drug delivery, poly(glycerol acrylate), poly(solketal acrylate), polymeric nanoparticles, posterior eye segment, vitreous barrier

## Abstract

Owing the complex anatomy and multiple physiological barriers, delivering drugs to the posterior segment of the eye remains challenging. While intravitreal injection can improve drug delivery, the diffusion of therapeutics through the vitreous humor and their uptake by the retina remain limited due to the anionic nature of the vitreous humor, attributed to its collagen and glycosaminoglycan content. Poly(solketal acrylate)‐based nanoparticles (PSA‐NPs) were developed via single‐emulsion solvent evaporation and nanoprecipitation techniques and loaded with Nile red (NR‐PSA‐NPs) as a hydrophobic drug model, enabling in vitro and ex vivo tracking via fluorescence microscopy. These NPs exhibit a neutral to negative surface charge, enhancing their distribution through the vitreous humor. Evaluating these NPs for drug delivery, cytotoxicity studies using retinal cells confirmed their excellent nontoxic profiles. Additionally, fluorescence microscopy and FACS analysis revealed a significant increase in the uptake of NR‐PSA‐NPs in ARPE‐19 cells (human retinal pigment epithelial cell line) compared to that of free dye. Moreover, incubation of NR‐PSA‐NPs with retinal organ cultures and their intravitreal injection into whole porcine eyes showed red fluorescence across all retinal layers, confirming successful uptake, distribution, and widespread retinal penetration, making these NPs promising carriers for delivering hydrophobic drugs to the posterior eye segment.

## Graphical abstract

Nile Red‐loaded poly(solketal acrylate) nanoparticles enable fluorescence tracking and exhibit nontoxic profiles in retinal cells. Enhanced cellular uptake and deep retinal penetration after intravitreal injection highlight their excellent potential for targeted ocular drug delivery to the posterior segment of the eye.

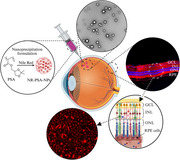

## 1 Introduction

Despite many efforts and advancements in biomedicine, drug delivery to the eye remains challenging because of the complex anatomy of the eye. Posterior eye diseases such as age‐related macular degeneration (AMD), diabetic retinopathy, retinal vein occlusion, retinitis pigmentosa, amongst others, that are associated with the most vision‐threatening conditions have adversely impacted the quality of life for many individuals globally [1, 2, 3]. Hence, improvement in effective treatment is a very important step for a better quality of life for millions of patients around the world [4]. There are several drug administration routes, such as topical administration, trans‐scleral delivery, subretinal, and intravitreal injections. Intravitreal injection is the most commonly used and preferred method in clinical practice for delivering drugs to the retina, offering effective local therapy with minimized systemic side effects, and is widely adopted for treating retinal diseases, since limited uptake or hindered penetration into the eye remain major drawbacks of other administration routes [1, 5, 6, 7, 8]. Although a higher drug concentration in the retina is achieved through intravitreal injection, followed by a decrease in systemic side effects [2], some major concerns remain unsolved in this strategy. The duration of action of freely administered drugs is limited by two factors: the retention time within the eye and the ocular clearance mechanisms, necessitating repeated injection, which may increase the risk of retinal detachment, infection and vitreous hemorrhage in patients [1, 9]. Additionally, the distribution of the drug through the vitreous humor barrier and its penetration to the retinal layers is still complicated. These challenges may be resolved by the implementation of nano drug delivery systems made from various materials offering properties, such as sustained release of the drug [8, 10, 11]. In addition, since the half‐life of the intravitreal drug is affected and related to its size, NPs with a larger size compared to the bare drug can prolong the retention time of the drug between three to six months within the intravitreal space [12, 13, 14, 15, 16].

Synthetic polymers with favorable mechanical, chemical, and/or degradation properties can be employed in ocular drug delivery systems [17, 18, 19]. Poly(ethylene glycol) [20, 21], poly(lactic‐co‐glycolic acid) [22, 23, 24, 25, 26, 27], and poly(caprolactone) [20] are the most common polymers used in intravitreal nanoparticles application for retinal delivery of hydrophobic therapeutics [28]. While biodegradable polymers like poly(lactic‐co‐glycolic acid) offer advantages due to their biodegradability, their degradation‐driven release often causes burst release of the drug [29]. In contrast, non‐biodegradable polymers can provide steadier, diffusion‐controlled release and help avoid sharp drug concentration peaks. For instance, poly(ethylene glycol) is a non‐biodegradable polymer used in ocular treatments enhancing corneal absorption of topical steroids and prolonging the half‐life of intravitreal therapeutics for sustained ocular drug delivery [28, 30, 31]. In this work, we have investigated nanoparticles based on poly(solketal acrylate) (PSA), which undergoes acid hydrolysis to poly(glycerol acrylate) (PGA) and subsequently to poly(acrylic acid) (PAA), and poly(vinyl alcohol) (PVA). PSA offers additional functionality due to the presence of acid‐labile solketal groups, enabling triggered drug release under acidic conditions. Both PVA and PAA, are of particular interest since they have already been used in ocular applications and several approved formulations have demonstrated that these polymers are well tolerated in the eye, supporting their suitability for ophthalmic use. Vitrasert, an intraocular implant containing PVA, remains in the eye after drug release [32]. PAA is another non‐biodegradable polymers that has high potential owing to its mucoadhesive properties. Its crosslinked form, known as carbomer, is widely used in ophthalmic gel formulations such as Viscotears [33]. A closely related derivative, polycarbophil, represents another crosslinked form of PAA and serves as the mucoadhesive component in the DuraSite ocular drug delivery system [34]. In addition, commercial products such as NyoGel and Pilogel also utilize such systems [35, 36]. However, PSA itself has being studied mainly as a polymer precursor to PGA [37, 38, 39] or in micellar systems [40, 41, 42]. In one study by Zhang et al., an amphiphilic block polymer comprising PSA and monomethoxy poly(ethylene glycol) was utilized for synthesizing potential pH‐responsive micelles with application in drug delivery [40]. Thus, in this work the potential of this polymer to form nanoparticles for ocular drug delivery is investigated. Solketal acrylate is generally polymerized via radical polymerization methods such as free radical polymerization [39, 43, 44, 45], reversible addition−fragmentation chain‐transfer (RAFT) polymerization [46], or standard atom transfer radical polymerization (ATRP) [38, 41, 42, 47] using high copper‐contents and/or high temperatures. Only one study reports on the use of single‐electron transfer living radical polymerization using Cu‐powder to avoid high catalyst loadings [37]. In this study, precisely defined PSA with different targeted degrees of polymerization (DP) were synthesized using supplemental activator and reducing agent atom transfer radical polymerization (SARA‐ATRP) using copper‐wire, enabling precise control over polymer architecture with fast reaction rates, low catalyst loading, and high end‐group fidelity. Additionally, the simple removal of copper wire at the end of the polymerization results in reduced levels of copper residues in the final products, which is ideal for drug delivery applications [48]. PSA was used to create both empty and Nile red (NR)‐loaded PSA‐ nanoparticles (NPs). NR was chosen as a hydrophobic drug model because it mimics hydrophobic compounds and exhibits strong red fluorescence in nonpolar environments, enabling precise localization and tracing of loaded nanoparticles in cells or tissues via fluorescence imaging. Owing to its pronounced solvatochromic behavior, NR remains nearly non‐fluorescent in aqueous media but becomes highly fluorescent when located in hydrophobic domains such as the nanoparticle core or, after release from its carrier, upon interaction with cytosolic lipids, hydrophobic pockets of proteins and cellular membranes [49, 50, 51, 52].

Due to the crucial role of NPs size in ocular drug delivery, two different nanoformulation methods of single emulsion solvent evaporation and nanoprecipitation were employed to assess their impact on the size and polydispersity index (PDI) of the resulting NPs. These techniques are commonly employed to produce polymeric NPs and encapsulate hydrophobic cargos. Additionally, cytotoxicity profiles and NPs uptake were evaluated using both the ARPE‐19 cell line, a human retinal pigment epithelium (RPE) cell line, and primary‐derived porcine Müller cells [53]. Müller cells are specialized radial glial cells spanning the entire thickness of the neural retina [54]. To evaluate the behavior of NR‐PSA‐NPs in a physiologically relevant setting, ex vivo porcine retinal organ cultures were used to assess their ability to overcome ocular barriers and reach the retina [55]. Initial studies with retinal punches confirmed penetration and uptake across key retinal layers (ganglion cell layer (GCL), inner nuclear layer (INL), and outer nuclear layer (ONL)). Subsequent experiments using dissected eyes (without vitreous humor) were performed to observe broader distribution, and finally, intact porcine eyes were used to test intravitreal injection and NP delivery through the vitreous barrier to the posterior segment. Thus, NR‐PSA‐NPs showed improved cellular uptake and efficient retinal penetration in ex vivo porcine models, revealing their potential as versatile carriers for hydrophobic drugs targeting the posterior eye segment.

## 2 Results and Discussion

### 2.1 Polymerization Studies

**FIGURE 1:**
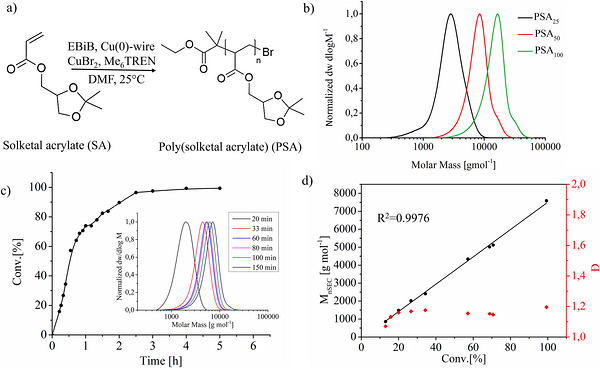
(a) Synthesis route of PSA by SARA‐ATRP. (b) SEC traces of PSA_25_, PSA_50_ and PSA_100_. (c) Polymerization screening by an aliquot method with SA (EBiB:SA = 50) with a fixed ratio of EBiB:CuBr_2_:Me_6_TREN = 1:0.05:0.18 with 5 cm Cu(0)‐wire. Conversion measured by ^1^H‐NMR spectroscopy and number‐average molar mass determined by SEC. d) Dependency of M_n_ and Ð on the conversion determined by SEC, showing a linear relationship between the molecular weight and conversion.

PSA was synthesized using solketal acrylate (SA) as monomer through SARA‐ATRP with three different target DP of 25, 50, and 100 resulting in DP_conv_ (DP based on exact initial weights and conversion) of 23, 46, and 90 (Figure 1 and Table 1). Solketal acrylate was synthesized according to a literature procedure (Figure S1) [45]. Due to better solubility of SA in DMF than in DMSO, DMF was chosen as a solvent for polymerization in this study, as already proven in other studies as a viable solvent for hydrophobic monomers [56, 57, 58]. The successful polymerization of SA demonstrates that SARA‐ATRP can be employed as a simple approach for producing materials for drug delivery applications. The controlled nature of this technique resulted in the successful production of homopolymers. According to size‐exclusion chromatography (SEC) results, by increasing the SA content in homopolymers, an increase in molar masses was observed from 3.3 to 7.2 and 13.1 kg mol^−1^, respectively (Figure 1b and Table 1). Based on the isolated polymer and given the high end‐group fidelity, the absolute molecular weights and degrees of polymerization of the respective polymers were calculated by ^1^H‐NMR spectroscopy and were in close agreement with the DP_conv_, yielding values of 3.9 (DP = 20), 8.8 (DP = 46), and 15.8 kg mol^−1^ (DP = 84), respectively (Figures S2–S4 and Table 1).

**TABLE 1:** Results of SA polymerization.

Entry	Name^a^	EBiB:SA^b^ (DP_conv_)^c^	Conversion (%)^d^	*M_n_*_, SEC,PSA_ (×10^3 ^g mol^−1^)^e^	*M_n_*_, NMR,PSA_ (×10^3 ^g mol^−1^)^f^ (DP)	Ð_PSA_ ^e^	*M_n_*_, NMR,PGA_ (×10^3 ^g mol^−1^)^f^ (DP)
1	PSA_25_	1:25 (23)	99	3.3	3.9 (20)	1.30	3.7 (24)
2	PSA_50_	1:50 (46)	>99	7.2	8.8 (46)	1.19	6.9 (46)
3	PSA_100_	1:100 (90)	98	13.1	15.8 (84)	1.21	13.1 (88)

^a^ Polymerizations were carried out at room temperature for 24 h with a fixed ratio of 1:0.05:0.18 of EBiB:CuBr_2_:Me_6_TREN with 5 cm Cu(0)‐wire.

^b^ Initial target DP as EBiB:SA.

^c^ DP_conv_ based on the actual weights and the conversion.

^d^ Calculated via ^1^H‐NMR spectroscopy (see Supporting Information for more information).

^e^ Molecular weight (*M_n_*) and Ð = *M_w_* /*M_n_* as determined via SEC in chloroform relative to polystyrene standards.

^f^ Calculated via ^1^H‐NMR spectroscopy (see Supporting Information for more information).

Kinetic measurements for SA polymerization (EBiB:SA = 50) were performed to verify the control of the polymerization (Figure 1c,d). A controlled polymerization was observed, resulting in low polydispersity of the isolated PSA samples (1.07≤*Đ*≤1.20) and further proved by the linear relationship between the molecular weight and conversion. Additionally, when conversion is plotted versus time, the polymerization rate can be analyzed. For kinetic analysis, a linear first‐order kinetic plot was constructed (Figure S9). According to the first section of this plot (t = 0–25 min), a slight initiation period is observed, attributed to the reduction of Cu(II) before the ATRP equilibrium is established [59]. The reaction rate constant (*k_p_*) was determined from the slope (0.021 min^−1^) and the initial EBiB concentration, yielding a value of 0.457 L (mol min)^−1^.

### 2.2 Hydrolysis Experiments

PSA is a hydrophobic polymer that can undergo hydrolysis under acidic conditions, leading to the deprotection of hydroxy groups. The hydrolysis process forms hydrophilic PGA that can be characterized by the two hydroxyl groups in each of its repeating units [47, 60, 61]. To deprotect PSA and study the characteristics of the resulting PGA, PS_50_ was treated with Amberlyst 15 in a microwave‐assisted reaction to facilitate hydrolysis without the use of hydrogen chloride. Following the reaction, aliquots of the hydrolyzed polymer (PGA_50_) were analyzed by ^1^H‐NMR and Fourier transform infrared (FTIR) spectroscopy to further examine its structural alterations. An absence of the two signals at δ = 1.35 and 1.28 ppm in the ^1^H‐NMR spectrum, corresponding to the two methyl groups in the ^1^H‐NMR spectrum of PSA_50_, is observed (Figures S6 and S8). Similar ^1^H‐NMR spectroscopic outcomes were detected for PGA_25_ and PGA_100_ (Figures S5 and S7). Additionally, the number of repeating units as determined by ^1^H‐NMR spectroscopy remained unchanged between PSA and the respective PGA and is still in close agreement with the DP_conv_ (Figures S5–S7; Table 1). FTIR analysis of PGA reveals the appearance of a novel band at 3366 cm^−1^, indicative of the hydroxyl groups present in the repeating unit of PGA_50_ (Figure S10). Notably, this signal is absent in the FTIR results of the original PSA_50_ polymer.

### 2.3 Nanoparticle Formulation

Polymers with higher molecular weights are commonly favored in drug delivery because they offer advantages in drug encapsulation, sustained release, and formulation stability. In addition, their larger molecular size and greater chain entanglement enhance their capacity to encapsulate and protect therapeutic agents, thereby improving both bioavailability [62] and overall therapeutic efficacy [63]. Thus, PSA_100_ was chosen for nanoparticle development using two common methods: single emulsion solvent evaporation and nanoprecipitation [64]. Because PSA_100_ is hydrophobic, using a stabilizer is essential in any nanoformulation method to produce stable NPs. Stabilizers prevent phase separation and improve emulsion stability by electrostatic and steric repulsion of NPs [65]. PVA is a hydrophilic, biocompatible polymer commonly used as a stabilizer because its temperature and pH stability, along with strong mechanical properties, make it ideal for pharmaceutical and biopharmaceutical encapsulations [66].

**FIGURE 2:**
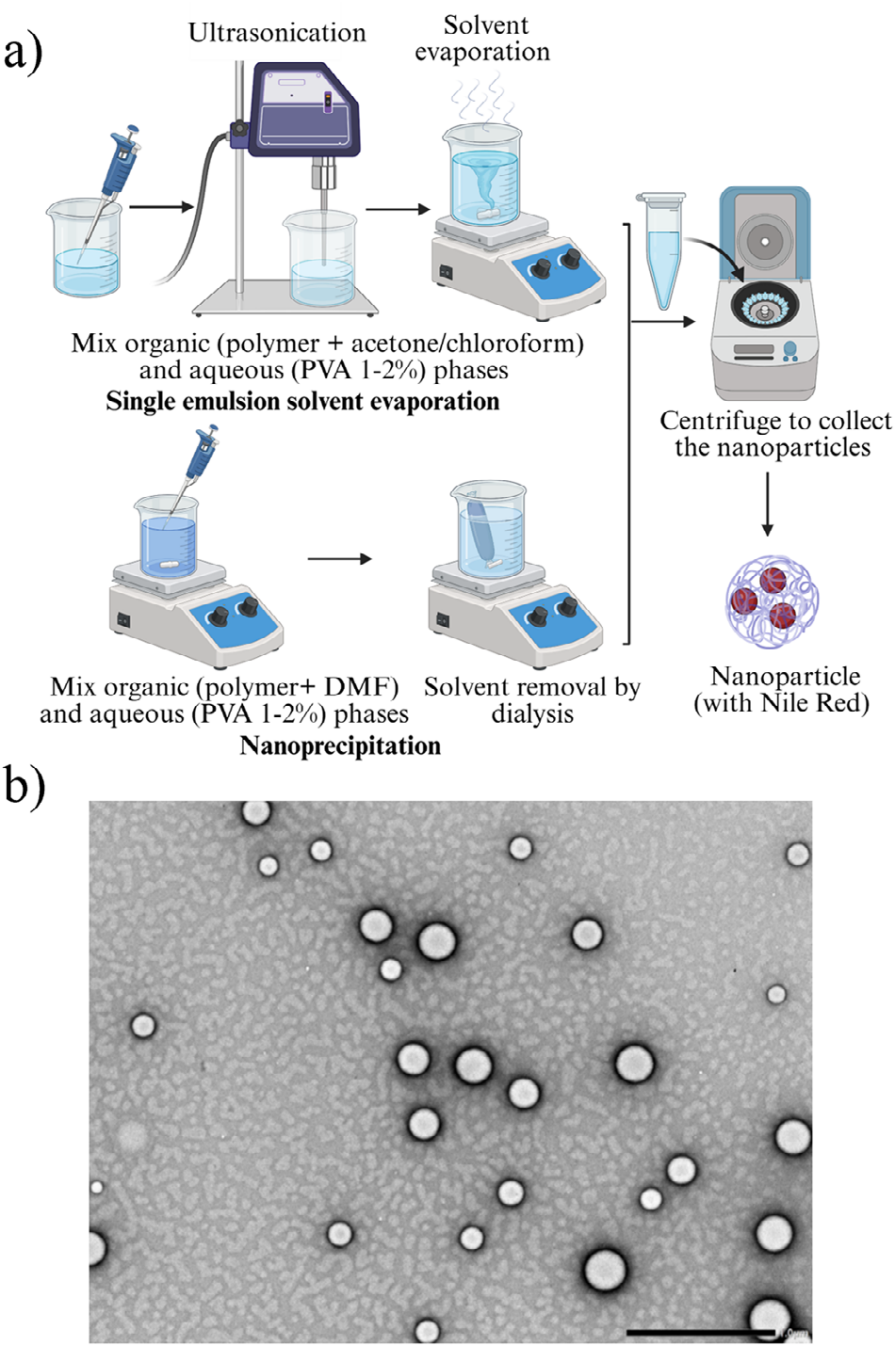
(a) Schematic representation of single‐emulsion solvent evaporation and nanoprecipitation methods. (b) TEM image of spherical NR‐PSA_100_‐NPs. Scale bar is set to 1 µm. Schematic images were created using Biorender.com.

The single emulsion oil‐in‐water (o/w) method, a common technique for emulsion formation, was chosen for solvent evaporation nanoformulation in this study (Figure 2a) [67]. For this, the volatile solvent dissolves the polymer and/or drug, and by further mixing the organic phase with an aqueous phase containing stabilizer (PVA), an emulsion is formed; after solvent evaporation, nanoparticles form and disperse within the aqueous phase [68, 69, 70]. Various factors such as polymer concentration, PVA molecular weight and concentration, sonication power and time, and aqueous phase volume were adjusted during NPs formulation to study their effects on size and PDI evaluated by dynamic light scattering (DLS) and Zeta potential (for some samples) determined by electrophoretic Light scattering (ELS) (Entries SE1‐12, Table S1). PSA_100_‐NPs prepared by this method ranged from 134 to 571 nm in size, with PDIs between 0.03 and 0.37 (Entries SE1‐12, Table S3). The smallest and most stable NPs (134 nm, PDI 0.09) were formed from formulations SE5 and SE9 (Entries SE5 and SE9, Table S3). The optimized sizes of NPs in SE5 and SE9 were caused by longer sonication time (20 min) and lower polymer concentration (3.46 mg mL^−1^), respectively.

Nanoprecipitation, also named solvent displacement, is another technique employed in this study (Figure 2a). It involves two miscible solvents: one dissolves the polymer and/ or drug, and the other acts as a non‐solvent. The rapid diffusion of the organic phase into the aqueous phase due to surface tension at the interface of the two phases leads to the formation of NPs with a narrow distribution [71, 72]. The non‐solvent phase includes the stabilizer to enhance NPs stability. Solvent removal is chosen by solvent type: acetone is evaporated overnight, while DMF is removed by dialysis with regular water changes [67, 72, 73]. Several factors affecting nanoprecipitation formulations were studied including polymer concentration, final suspension volume, aqueous phase type (water, PVA, PBS), PVA concentration, organic phase addition rate, aqueous phase temperature (room temperature vs. 80°C), mixing speed (1000 vs. 1500 rpm), and sonication (Table S2). Nanoprecipitation formulations produced NPs with sizes between 154–763 nm and with PDIs of 0.05–0.65 (Entries P1‐P16, Table S4). Amongst all formulations, P7 (171 ± 1.38 nm, PDI 0.06) was preferred because of its easier synthesis procedure and lower PVA amount (Entry P7, Table S4).

In summary, both methods are able to produce uniformly sized NPs with narrow polydispersities, highly dependent on variation and optimization of production parameters. Nonetheless, the ease of nanoprecipitation, especially for formulation P7, made it the preferred formulation in this study. Hence this method was also used to create NR‐PSA_100_‐NPs by adding NR to the polymer solution. According to the DLS results (Entry 2, Table 2), uniform NPs with 176 nm size and a PDI of 0.02 were formed. Compared to the empty NPs, the loaded NPs exhibited slight increase in size, accompanied by comparable PDI value (Entries 1 and 2, Table 2). The zeta potentials of both empty PSA_100_‐NPs and NR‐PSA_100_‐NPs were measured, yielding values of −5.7 mV and −4.5 mV, respectively. Due to composition of the vitreous humor mainly consisting of water and glycosaminoglycans, especially hyaluronic acid, producing NPs with a neutral to negative zeta potential is the key for better diffusion and retinal penetration. The observed negative surface charges are advantageous, as they are expected to promote enhanced mobility and distribution of the particles within the vitreous humor [10, 74]. Transmission electron microscopy (TEM) images were captured to investigate the morphology, size, and shape of the empty PSA_100_‐NPs as well as NR‐PSA_100_‐NPs. According to these images (Figure 2b; Figure S13), both empty PSA_100_‐NPs and NR‐PSA_100_‐NPs were spherical and well‐dispersed, with minimal aggregation. ImageJ analysis of two TEM images of NR‐PSA_100_‐NPs indicated mean particle sizes of 122 and 168 nm (Figure S13a,b), while analysis of empty PSA_100_‐NPs showed lower mean sizes of 57 and 71 nm (Figure S13c,d).

**TABLE 2:** Size, PDI, and zeta potential of empty PSA_100_‐NPs and NR‐PSA_100_‐NPs produced by nanoprecipitation method P7 determined by DLS and ELS at 25°C.

Entry	Formulation	Size (nm)	PDI	Zeta potential (mV)
1	Empty PSA_100_‐NPs	171	0.06	−5.7
2	NR‐PSA_100_‐NPs	176	0.02	−4.5

**FIGURE 3:**
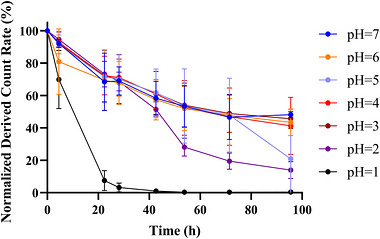
Disassembly of empty PSA_100_‐NPs at different pH values plotted as normalized derived mean count rate (%) resembling number of NPs versus time (up to 95 h) at a pH range of 1–7.

Since the release kinetics of the cargo from the core of the PSA_100_‐NPs can be affected by various factors such as hydrolysis, disintegration, and degradation, the stability of empty PSA_100_‐NPs in a cell‐free environment was screened. The derived mean count rate of NPs, as well as their size and PDI, were measured using DLS at varying pH values (1–7) at different time points (up to 95 h) while shaking at 37°C in an incubator. At the beginning of the measurement (t_0_), the NPs should show 100% derived mean count rate; however, as soon as the NPs start to shake and incubate at 37°C, a change in the number of NPs present in the suspension will occur, which is detected as a decrease in the derived mean count rate. As shown in Figure 3, NPs at pH 1 and 2 and partially at pH 3 were hydrolyzed faster under acidic conditions compared to the other pH values due to their significant increase in size since PSA is converted to PGA at these pH values. A general disintegration of the NPs occurs due to the decrease in the number of NPs and consequently the derived mean count rate for all tested pH values. Additionally, further insights can be obtained by analyzing the NP sizes using both Z‐average and number‐based measurements. According to the size plot based on the Z average (Figure S11), the size of the NPs incubated at pH values 3–7 remained constant while maintaining similar PDI. However, for NPs incubated at pH values 1 and 2, size is highly influenced by the formation of aggregates (supported by the increased PDI values) after 5 h of incubation. This high influence was attributed to the higher scattering of larger particles measured by DLS. In contrast, the mean number plot reports consistent NP size across all pH values, which can be representative of the higher population of NPs maintaining their original size, while a smaller subpopulation can form aggregates, especially at lower pH values (Figure S12). In conclusion, the disintegration of NPs can lead to sustained release of the drug over a longer period of time, which is advantageous for drug delivery to the posterior segment of the eye.

### 2.4 In Vitro Experiments

In vitro cell viability evaluation of PSA_100_‐NPs and PGA homopolymers was conducted using 3‐(4,5‐dimethylthiazol‐2‐yl)‐5‐(3‐carboxymethoxyphenyl)‐2‐(4‐sulfophenyl)‐2H tetrazolium salt (MTS) and crystal violet (CV) assays with two retinal cell models (ARPE‐19 cell line and primary derived porcine Müller cells). ARPE‐19, a human RPE cell line, is a commonly employed in vitro model as it mimics key features of RPE dysfunction linked to ocular diseases like AMD [75]. Müller cells, the main glial cells of the retina, are essential for retinal homeostasis, and their dysfunction contributes to inflammation and vascular abnormalities [54].

**FIGURE 4:**
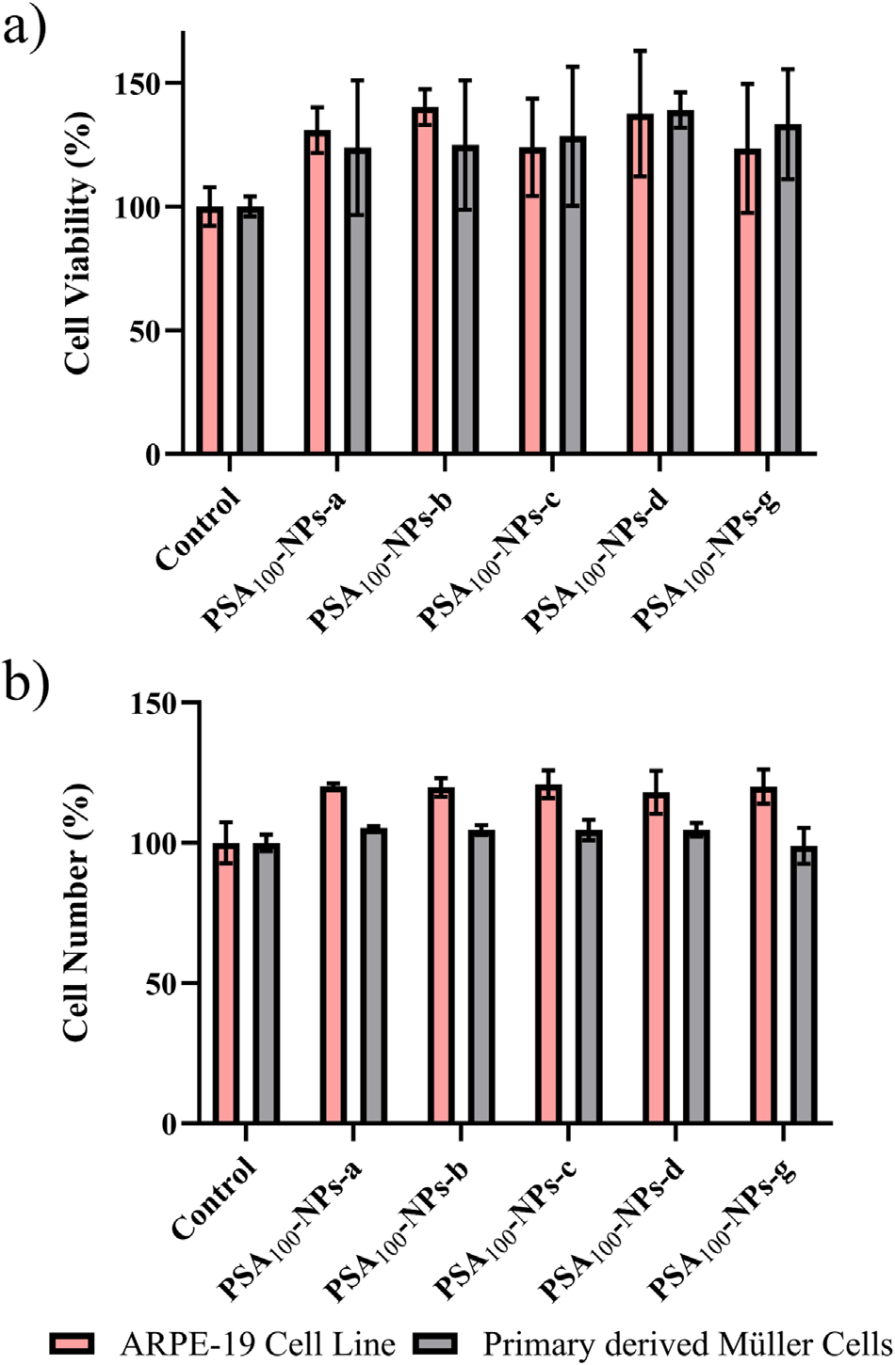
MTS (a) and CV (b) assays were conducted using varying dilutions of PSA_100_‐NPs with the ARPE‐19 cell line and primary‐derived Müller cells to assess cell viability and cell number, respectively. The culture duration for each assay was 24 h.

The MTS assay was used to assess the cytotoxicity profiles of the NPs [76]. Sufficient cell viability was demonstrated by the results of PSA_100_‐NPs (formed from nanoprecipitation formulation) at all concentrations (0.1 to 1000 µg mL^−1^ initial polymer concentration) after 24 and 48 h of incubation in both ARPE‐19 and primary‐derived Müller cells, implying an adequate level of cell viability (Figure 4a; Figure S14). The crystal violet (CV) assay was used to quantify and differentiate living cells that remain attached to the plate [77]. No notable disparity in cell number was observed between the PSA_100_‐NPs treated groups and the control with ARPE‐19 and primary‐derived Müller cells (Figure 4a). Similar results were obtained for 48 h of incubations (Figure S14).

To evaluate the cytotoxicity of PGA, which is a product of PSA hydrolysis, the synthesized PGA polymers (PGA_25_, PGA_50_, and PGA_100_) were incubated with ARPE‐19 cells at their highest concentration (1000 µg mL^−1^) for 24 h. According to their MTS results, ≈80% cell viability was reported for the polymer. Furthermore, the CV assay also showed up to ≈80% cell density compared to the control group, which falls within an acceptable range for such a high polymer concentration (Figure S15).

To assess the in vitro uptake of PSA_100_‐NPs, NR‐PSA_100_‐NPs were used in various experiments. NR, as a hydrophobic fluorescent dye, was selected as a model compound to enable the visualization of nanoparticle localization within cells and tissues through fluorescence imaging. This choice is particularly relevant as many drugs used in ophthalmology are hydrophobic, making NR a suitable model for mimicking drug behavior in ocular delivery systems. To determine whether these NPs could be taken up by cells, ARPE‐19 and primary‐derived Müller cells were incubated for 24 h with three different treatments: NR‐PSA_100_‐NPs‐c (0.6 mg mL^−1^ based on initial polymer concentration), free NR, and empty PSA_100_‐NPs. After incubation, fluorescence images were captured to assess the NP uptake in the cells. In both ARPE‐19 and primary‐derived Müller cells, free NR (the same amount as used for NPs formulation) could not be dissolved and internalized into cells properly due to low solubility, thus showing no red fluorescence (Figures S16 and S17). When using NR‐PSA_100_‐NPs, a distinct strong fluorescence from NR was observed (Figures S16 and S17). A significantly higher fluorescence was detected in cells treated with NR‐PSA_100_‐NPs than in cells treated with free NR, suggesting the successful function of PSA_100_‐NPs as drug delivery vehicles (Figure S18). Among the treated cell types, the ARPE‐19 cells showed stronger fluorescence than primary Müller cells when treated with NR‐PSA_100_‐NPs. In addition, bright‐field images show a normal morphology of the cells after incubation with NPs (Figures S16 and S17).

To further investigate the localization of NPs within the cells and their internalization route, LysoBrite Green was co‐transfected together with the NR‐loaded NPs. ARPE‐19 cells were incubated with three dilutions of NR‐PSA_100_‐NPs (NR‐PSA_100_‐NPs‐a to c) for 24 h. The lysosomes of NR‐PSA_100_‐NP‐treated cells were stained with LysoBrite Green to localize the lysosomes in the cells.

**FIGURE 5:**
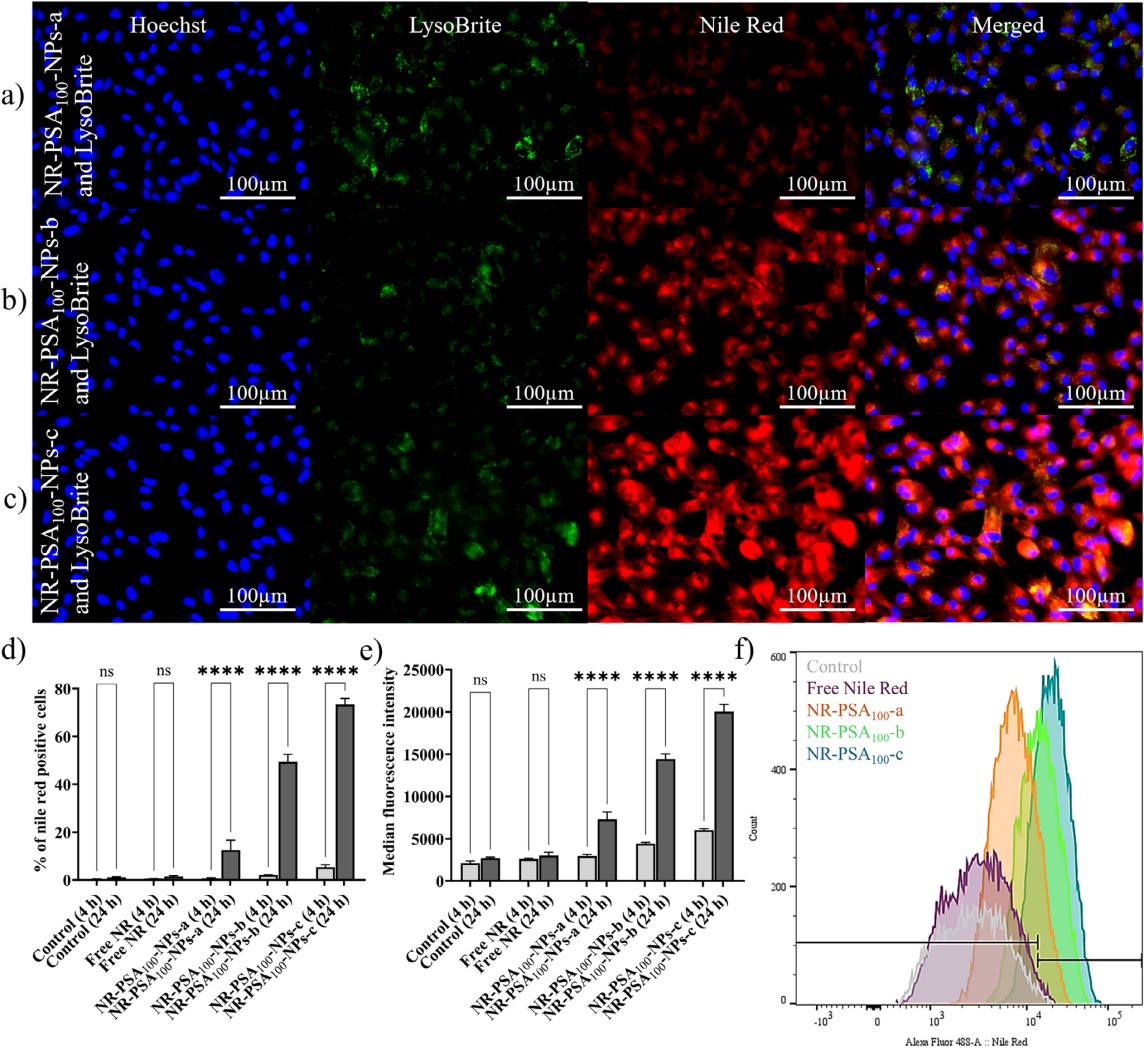
ARPE‐19 cells were subjected to Hoechst and LysoBrite Green staining after incubation with (a) NR‐PSA_100_‐NPs‐a, (b) NR‐PSA_100_‐NPs‐b, and (c) NR‐PSA_100_‐NPs‐c for 24 h. Fluorescence images (40X magnification) using blue (Hoechst), green (LysoBrite Green), and red (Nile Red) channels were taken from samples, and a scale bar of 100 µm was included for reference. The bar graphs show summary data of flow‐cytometry analysis (d) the percentage of NR positive cells and (e) the median NR fluorescence intensity after 4 vs. 24 h incubation with treatments at 37°C (*n* = 3 ± SD). The histograms represent the flow cytometry results from treatments with control, free NR, and NR‐PAS_100_‐NPs after 24 h incubation at 37°C (f). Statistical significance was determined using one‐way ANOVA followed by Tukey's post‐hoc test: * for *p* ≤ 0.05, ** for *p* ≤ 0.01, *** for *p* ≤ 0.001 and **** for *p* ≤ 0.0001.

NR‐PSA_100_‐NPs showed a concentration‐dependent uptake, and as the initial polymer concentration of NR‐PSA_100_‐NPs (with a fixed polymer: NR ratio) increased from 0.1 to 0.6 mg mL^−1^ in NR‐PSA_100_‐NPs‐a to c samples, respectively, and thus with a higher NR content, stronger NR fluorescence intensity is observed in fluorescence microscopy images (Figure 5). As the concentration of NPs increased, a decrease in green fluorescence from LysoBrite Green was observed, possibly due to competition in cellular uptake mechanisms. The images of the NR‐PSA_100_‐NP‐treated groups showed diffused red fluorescence, indicating a distribution within the cytoplasm of the cell, whereas only punctate green fluorescence from LysoBrite Green was visible in the lysosomes, suggesting that the NPs were likely internalized and present within all cell compartments, and trafficking for lysosomal degradation did not play a major role (Figure S19).

Flow cytometry was used to quantify cellular uptake by measuring the red fluorescence from NR in the treated cells and reporting the proportion of stained cells, while the median fluorescence intensity showed the average dye uptake per cell. ARPE‐19 cells were incubated under the following reaction conditions: control, free NR without polymeric carrier, NR‐PSA_100_‐NPs‐a, ‐b, and ‐c for either 4 or 24 h at 37°C. ARPE‐19 cells exposed to NR‐PSA_100_ NPs showed on average 6–8 fold significantly higher NR positive cell percentages and 3–4.5 fold higher median fluorescence intensities than those in the control (*p* < 0.0001) and 3–4 and 2–3 fold higher values, respectively, than free NR groups (*p* < 0.0001) (Figure 5; Figure S20). The results confirm the ability of the polymer carrier to deliver this hydrophobic drug model. In addition, the uptake of NR‐PSA_100_‐NPs was both concentration‐ and time‐dependent, with increasing median fluorescence intensity as NR content increased (NR‐PSA_100_‐NPs‐a < NR‐PSA_100_‐NPs‐b < NR‐PSA_100_‐NPs‐c) (Figure 5d–f) and 1.3–1.5 fold significantly greater NR positivity and fluorescence after 24 h incubation compared to 4 h (Figure 5d,e).

To further investigate the uptake mechanism of our NPs, the cells were incubated for 4 h at either 4°C or 37°C (Figure S21). As proposed by Snipstad et al. [78], endocytosis is largely suppressed at 4°C; thus, this comparison distinguishes energy‐dependent processes from alternative pathways such as contact‐mediated internalization. While cells treated with NPs, showed an average 1.8 fold greater NR positivity and 1.3 fold higher median fluorescence than controls, no significant difference was observed between the 4°C and 37°C groups, suggesting uptake is not solely reliant on endocytosis (Figure S21). It should also be noted that the overall uptake after 4 h was still far lower than that after 24 h incubation, indicating that an extended incubation duration also increases the contact time, enhancing internalization.

### 2.5 Ex Vivo Experiments

**FIGURE 6:**
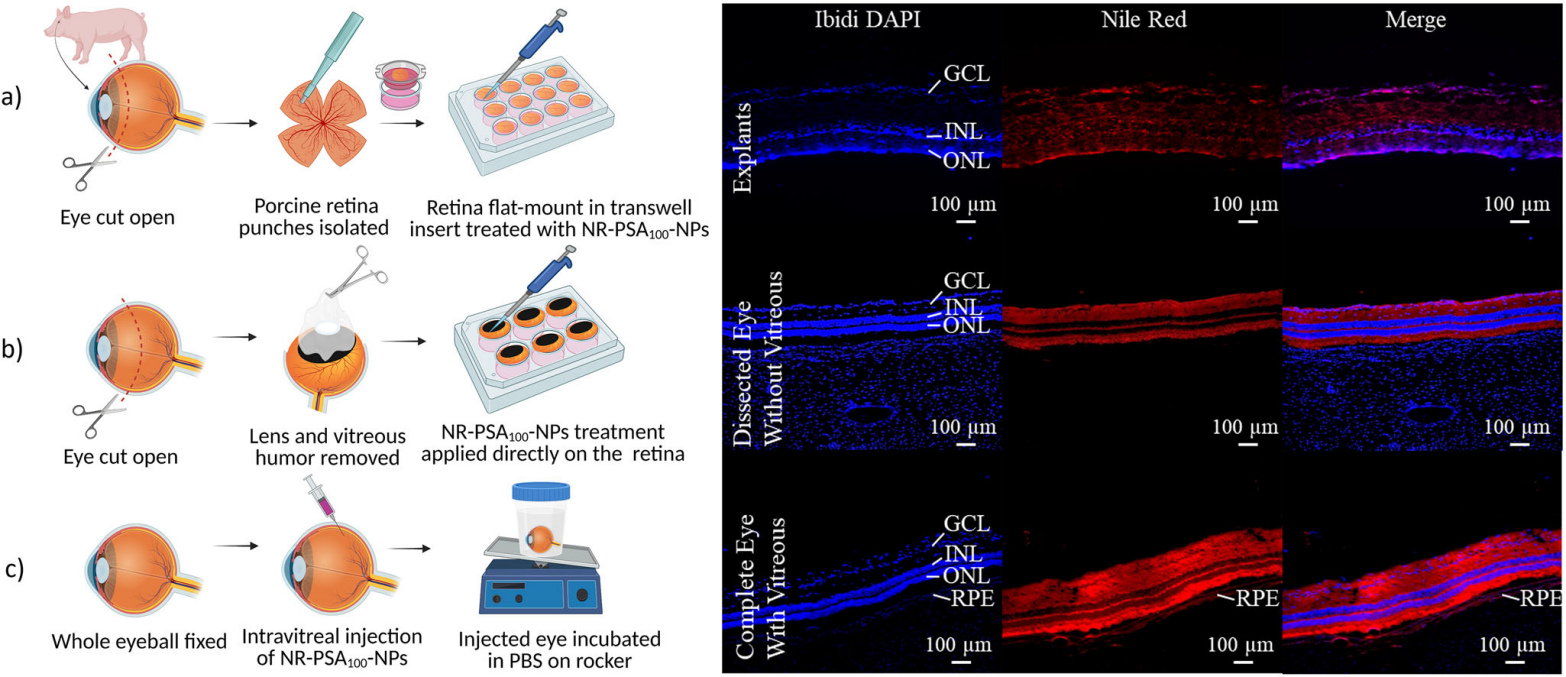
Schematic and fluorescence microscopy images of (a) porcine retina explants, (b) dissected porcine eye without vitreous, and (c) complete porcine eye treated with NR‐PSA_100_‐NPs‐c after 24 h of incubation. Ex vivo cultures were imaged using blue and red channels (for DAPI and NR fluorescence, respectively). A scale bar of 100 µm was included for reference. Schematic images were created with Biorender.com.

After assessing NR‐PSA_100_‐NPs uptake in vitro, NP uptake was assessed in ex vivo porcine retinal organ cultures. This approach provides deeper insight into their behavior in a more physiologically relevant setting. The porcine eye is an advantageous model in ophthalmic research due to its close anatomical and physiological resemblance to the human eye [79, 80]. Its size, dimensions [81], and the volumes of aqueous humor and vitreous body [82, 83] closely match those of humans. In addition, the porcine retina offers functional and structural features comparable to those of the human retina, including a similar thickness (≈300 µm vs. 310 µm in porcine and humans retinas, respectively [84]), making it especially suitable for studying eye diseases and disorders [85]. To evaluate the successful uptake and distribution of NR‐PSA_100_‐NPs within the retina, a systematic experimental approach was used. Initially, retinal punches were prepared to determine whether the NPs could penetrate and be internalized by retinal tissue. Owing to the hydrophobic nature of free and unencapsulated NR, it does not dissolve in the retinal medium; thus, it was filtered to remove any insoluble and undissolved solid particles from the treatment medium. NR‐PSA_100_‐NPs‐c showed red fluorescence throughout the entire thickness of the retina punches, unlike free NR, highlighting the essential role of PSA_100_‐NPs in encapsulating and delivering this hydrophobic cargo to the pig eye's posterior segment. (Figure 6a; Figure S22). Following confirmation of effective penetration and distribution of NR‐PSA_100_‐NPs across the full thickness of the retina, the study proceeded to a more detailed investigation. Given that retinal punches allow access from both sides within the inserts, the gradual distribution of the compounds across the retinal layers was assessed by uptake from the ganglion cell layer (GCL) and subsequently approaching to the inner nuclear layer (INL) and outer nuclear layer (ONL) was performed (Figure 6b). Porcine eyes were dissected, the vitreous humor was removed, and the retina remained intact. The red fluorescence of NR‐PSA_100_‐NPs was distributed from ganglion cells to inner and outer nuclear layers, and finally through the photoreceptor outer segment, demonstrating effective uptake and uniform distribution across all retina layers (for complete sets of images, see Figure S23).

To further investigate transit through the vitreous and subsequent cellular uptake and distribution within the retinal layers and RPE cells, an ex vivo model with intact porcine eyes was used, and an intravitreal injection of NR‐PSA_100_‐NPs was performed on the model. The results demonstrated that the developed PSA‐NPs successfully diffused through the vitreous body, reached the retinal tissue, and were efficiently internalized and distributed through the thickness of the retina, ultimately reaching the RPE cells (Figure 6c; Figure S24). These results establish these NPs as a clinically relevant platform for drug delivery to the retina after intravitreal injection, paving the way for their application in ophthalmic treatment.

## 3 Conclusion

In this study, PSA was successfully synthesized via SARA‐ATRP, yielding well‐defined polymers with controlled molecular weights and low polydispersities. Both nanoprecipitation and single emulsion solvent evaporation methods produced PSA_100_ nanoparticles with comparable size and PDI, but nanoprecipitation was preferred for its simplicity in generating drug‐delivery‐suitable NPs. Cytotoxicity assessments on ARPE‐19 and primary‐derived Müller cells confirmed excellent cell viability, even in the most concentrated NP suspension (1 mg mL^−1^ based on initial polymer concentration), supporting the safety of these materials for ocular treatments. NR‐loaded PSA_100_‐NP showed enhanced cellular uptake and distribution through the whole cell cytoplasm compared to free NR, likely due to improved solubility and stabilization of the hydrophobic dye within PSA_100_‐NPs and an uptake mechanism not only relying on endocytosis, highlighting their potential for delivering hydrophobic cargo. NR‐PSA_100_‐NP also showed a concentration‐ and time‐dependent uptake with higher NR content and longer incubation time, leading to higher median fluorescence intensity. Ex vivo porcine retinal models were employed to study NP behavior in a physiologically relevant setting. Retinal punches and dissected eyes without the vitreous humor demonstrated effective NP penetration into the key retinal layers (GCL, INL, ONL). Moreover, intravitreal injection into intact porcine eyes showed the advantages of negatively‐charged to neutral NPs to enable diffusion through the negatively charged vitreous humor to reach the retina and leading to retinal distribution within all retinal layers up to the RPE. These results collectively demonstrate the strong potential of poly(solketal acrylate) nanoparticles as an effective ocular drug delivery platform, especially for the posterior segment of the eye. Future work could explore loading these nanoparticles with ocular‐specific drugs and comparing the therapeutic effects of free versus encapsulated formulations in retinal disease models (in vitro and ex vivo). Moreover, extending ex vivo experiments to in vivo models would provide valuable information on the translational potential and practical applications of these nanoparticles.

## Funding

F.A. and Y.P.H. are thankful for funding by the Federal Ministry of Education and Research (BMBF) and the Baden‐Württemberg Ministry of Science as part of the Excellence Strategy of the German Federal and State Governments. F.A. is thankful for funding from the Dr. Leni Schöninger Foundation.

## Conflicts of Interest

The authors declare no conflicts of interest.

## Supporting information




**Supporting File**: marc70168‐sup‐0001‐SuppMat.pdf.

## Data Availability

The data that support the findings of this study are available in the supplementary material of this article.
