# Supplementary material for: Ex Vivo Evaluation of Poly(Solketal Acrylate) Nanoparticles for Intravitreal Drug Delivery to the Posterior Eye Segment
Source: Macromol Rapid Commun. 2025 Dec 12;47(14):e00788. doi: 10.1002/marc.202500788 (PMC13384798; doi:10.1002/marc.202500788)
Supplement: Supplementary file 1 — Supporting File: marc70168‐sup‐0001‐SuppMat.pdf. [file MARC-47-e00788-s001.pdf]

Supporting Information

Ex Vivo Evaluation of Poly(solketal acrylate) Nanoparticles for Intravitreal Drug Delivery to the Posterior Eye Segment

Yasaman Pourdakheli Hamedani, José Hurst, Malte Ritter, Philipp Weingarten, Julia Skokowa, Sven Schnichels, and Friederike Adams*

1. Materials and Methods

The reactions were conducted in a glovebox filled with argon (MBraun EcoLab). Standard Schlenkline technique was used for degassing of solvents and liquid chemicals for polymerization reactions. Tris[2-(dimethylamino)ethyl]amine (Me_6TREN) was supplied from Tokyo Chemical Industry Co., Ltd. (TCI, Tokyo, Japan). Triethylamine (99%) was purchased from CHEMSOLUTE®. Solketal (DL-1,2-isopropylidene glycerol 97%), $CuBr_2$ powder, and Ethyl α-bromoisobutyrate (EBiB), Amberlyst® 15 were provided from Sigma-Aldrich (Taufkirchen, Germany). Extra dry DMF, acryloyl chloride (96 %, stabilized with 400 ppm phenothiazine, Alfa Aesar™), Cu(0)-wire (gauge 0.25 mm, Alfa Aesar™ Puratronic™) were obtained from ThermoFischer GmbH (Kandel, Germany). A Cu(0)-wire wrapped magnetic stirring bar was submerged in HCl and rinsed with water (x2) and acetone (x2) followed by air-drying for purification. Sterile disposable PES syringe filters (0.45 µM) were purchased from Fischer Scientific. Neurobasal media, NucBlue Live Cell stain ReadyProbes, N2-supplement, B27-supplement, Gentamycin (10 mg mL^{-1}), Penicillin streptomycin, Dulbecco's Phosphate Buffer Saline (DPBS 1X), and Trypsin EDTA were purchased from Thermo Fisher Scientific (Darmstadt, Germany). BDNF (brain-derived neurotrophic factor human), CNTF (ciliary neurotrophic factor human), Fluorsave, and PFA (paraformaldehyde) were sourced from Merck (Darmstadt, Germany). 8 mm dermal punches were provided from Pfm medical AG (Koeln, Germany). Petri dishes were acquired from Greiner Bio-One (Frickenhausen, Germany). 12-Well plates were obtained from Nerbe plus (Winsen/Luhe, Germany). Disposable safety scalpel, forceps, eye-scissors and spatulas were purchased from Aesculap AG (Tuttlingen, Germany) and Geuder AG (Heidelberg), respectively. LysoBrite™ Green was provided from AAT Bioquest. Dichloromethane 99.5%, Chloroform 99% and Methanol 99% were purchased from Cal Roth GmbH (Karlsruhe, Germany). Ethanol 99% was purchased from SAV Liquid Production GmbH (Inn, Germany). IR spectroscopy was performed using Bruker Alpha

Platinum ATR. The received IR spectra were evaluated by the program Opus. The SONOPULS ultrasonic homogenizer was provided from BANDELIN electronic GmbH & Co. KG (Berlin, Germany).

For purification of nanoparticles (NPs) *via* dialysis method, molecular porous membrane tubing (Spectrum™ Spectra/Por™ 3 RC dialysis membrane) with a MWCO of 3.5 kDa and 9.3 mL cm^{-1} volume-length ratio was obtained from Spectrum Laboratories, Inc. (America). To dissolve samples for ^{1}H-NMR spectroscopy, deuterated solvents were provided from Sigma-Aldrich, Deutero, or Eurisotop. ^{1}H-NMR measurements were performed on a Bruker Avance III 400 or Bruker Avance III HD 700 NMR spectrometer (^{1}H-NMR spectroscopic chemical shifts δ are reported in ppm). δ (1H) is calibrated to the residual proton signal of the deuterated solvent. Size exclusion chromatography was used for the determination of relative average number and weight average molecular weights and polydispersity (M_n, M_w, and Đ). Polystyrene standards were utilized for measuring relative molar masses. The device contains a PSS SDV 5 µm 8*50 mm guard column with three PSS SDV 100,000 Å 5 µm 8*50 mm columns heated to 40 °C that are connected to a refractive index (RI) detector Agilent 1200 Series G1362A.

A Zetasizer Ultra Red (Malvern Instruments, Malvern, UK) was used to perform dynamic light scattering (DLS) measurements to determine the size (hydrodynamic diameter, $Đ_h$ as the z-average or as a number-based intensity) and polydispersity index (PDI). Samples were characterized using disposable cuvettes (DTS0012) with a backscattering collection angle. The measurement setting included equilibration at 25 °C for 120 seconds.

To conduct the biocompatibility studies, the ARPE-19 cell line was obtained from the American Type Culture Collection (Manassas, VA, USA). The culture medium in all experiments was Dulbecco's modified Eagle's medium containing 10% fetal bovine serum, streptomycin sulfate (100 µg mL^{-1}), penicillin G (100 U mL^{-1}), and L-glutamine (4 mM). Cells were maintained in an incubator at 37 °C and 5% CO_2.

To isolate the primary-derived Müller cells, porcine eyes were obtained from a local abattoir and immediately (maximum 3 hours after being sacrificed) transported to the lab while maintaining eyes at 4 °C. Immediately after (within 1 h of arrival), eyes were cleaned and sanitized by submerging in 70% ethanol for 5 minutes followed by two times washing using PBS. Then each eye was opened up by means of a scalpel under sterile conditions, followed by removal of the lens, cornea, and vitreous humor. Then the retinas were collected and digested using 1% papain (Thermo Fisher Scientific, Karlsruhe, Germany) and DNase I (AppliChem, Darmstadt, Germany) for 15 minutes while incubated in a thermo shaker (37 °C, 900 rpm, VWR, Darmstadt, Germany). The digested retinas were then centrifuged for 5 minutes to form

a pellet. The cell pellet was resuspended in the before mentioned medium and transferred into a 6 well plate (Thermo Fisher Scientific, Karlsruhe, Germany) coated with 0.1% gelatin. Then the medium was replaced regularly until the primary derived Müller cells were confluent. After passaging cells in freshly coated flasks for three times, a pure culture of cells was obtained. [1, 2] Cells were not utilized after passage number five.

Synthesis of solketal acrylate (SA)

To synthesize the SA monomer[3], dichloromethane (130 mL), DL-1,2-isopropylidene glycerol (solketal, 26.0 mL, 209 mmol, 1.0 eq) and triethylamine (38.0 mL, 274 mmol, 1.13 eq) were mixed in a heat-fried flask filled with argon and the reaction mixture was cooled down to 0 °C. Then acryloyl chloride (16.9 mL, 209 g mol^{-1}, 1.0 eq) was added to the mixture dropwise while stirring for 30 minutes. Then the stirring was continued overnight and afterwards the liquid phase was separated from the precipitate *via* filtering and was washed with water, 10% sodium hydrogen carbonate solution and saturated saline solution and dried over sodium sulphate. After filtration and distilling off the solvent, SA was mixed with a tip of spatula hydroquinone to prevent spontaneous polymerization. Then it was purified by vacuum distillation at 47 °C and 0.078 mbar and the desired product was received. Final product (24.5 g, 131 mmol, 63% yield) was obtained as a colorless solvent and was analyzed *via* ^{1}H-NMR spectroscopy.

Homopolymerization procedure

Polymerizations were performed at room temperature in an argon-filled glovebox. First, the $CuBr_2$ powder (2.47 mg, 0.011 mmol, 0.05 eq.) and Me_6TREN (10.7 µL, 0.039 mmol, 0.18 eq.) were dissolved in DMF (2.25 mL) in a glass vial (pre-dried by heating). Then the respective amount of SA monomer (25, 50, or 100 eq.) was added to the vial together with a pre-cleaned magnetic stirrer (wrapped with Cu(0)-wire (5 cm)). Finally, the polymerizations were started by the addition of the EBiB initiator (32.6 µL, 0.22 mmol, 1.0 eq.). After 24 hours of stirring, the polymerization was stopped through the removal of the magnetic stir bar and copper wire. To investigate the monomer conversion and molar mass analysis *via* ^{1}H-NMR spectroscopy and SEC, respectively, aliquots of the polymerization mixture were taken. For SEC analysis, aliquots were purified using filtration over aluminum oxide with chloroform (HPLC grade). For monomer conversion using ^{1}H-NMR spectroscopy, the acrylate signal of the monomer was compared with a proton signal occurring in both monomer and polymer signals. The crude product was first dissolved in DCM and then purified by filtration over aluminum oxide and

precipitated in pre-cooled hexane. Obtained polymers were dried in vacuo and characterized by ^{1}H-NMR spectroscopy and SEC.

Kinetic measurements

For kinetic measurement, the polymerization of PSA_{50} was repeated one more time, as described previously. This time, regular aliquots of the polymerization mixture were taken at certain time intervals and were characterized by ^{1}H-NMR spectroscopy and SEC for conversion measurements and molar mass determination, respectively. The SEC and ^{1}H-NMR samples were prepared, handled, and analyzed same way as described previously.

PGA synthesis

PSA (100 mg), chloroform (2 mL), methanol (2 mL), and amberlyst 15 (tip of spatula) were transferred to a microwave tube and placed in the microwave for 20 min at 60°C. Then the solution was transferred to a flask and dried under reduced pressure. The final product was analyzed *via* ^{1}H-NMR and FTIR spectroscopy.

Nanoparticle synthesis by the single emulsion solvent evaporation method

To synthesize NPs from PSA_{100}, different amounts of polymer were first dissolved in DCM (1 mL) to prepare various polymer concentrations from 2-12.5 mg mL^{-1}. Then the organic phase containing dissolved polymer was mixed with the aqueous phase (2.5-10 mL) containing PVA (13000-23000 or 146000-186000 g mol^{-1}, 0.5-2 wt %) and subjected to agitation *via* a probe sonicator (50-100% power) for a specified duration (ranging from 1 to 20 minutes). After sonication, the NPs suspension was stirred overnight for solvent evaporation at room temperature at 500 rpm. Size and PDI of the NPs were measured by DLS.

Table S1. Formulation parameters of empty PSA_{100}-NPs formed by the single emulsion solvent evaporation method.

#	Polymer concentration (mg mL^{-1})	PVA volume (mL)	PVA M_w (g mol^{-1})	Final polymer concentration (mg mL^{-1})	PVA concentration (w/w %)	Sonication power (%)	Sonication duration (min)
SE1	12.5	10	13000 – 23000	1.25	0.5	50	1
SE2	12.5	10	13000 – 23000	1.25	0.5	80	1
SE3	12.5	10	13000 – 23000	1.25	1	100	10
SE4	12.5	5	13000 – 23000	2.5	1	95	10
SE5	6.25	5	13000 – 23000	1.25	1	95	20
SE6	6.25	5	13000 – 23000	1.25	1	50	20
SE7	6.25	5	146000 – 186000	1.25	1	95	20
SE8	6.25	5	13000 – 23000	1.25	2	100	20
SE9	3.46	5	13000 – 23000	0.69	1	95	10
SE10	2	5	13000 – 23000	0.4	1	100	10
SE11	2	5	13000 – 23000	0.4	0.5	100	10
SE12	2	2.5	13000 – 23000	0.8	1	100	10

Nanoparticle synthesis by the nanoprecipitation method

To synthesize NPs from PSA_{100}, an organic phase was prepared by dissolving different amounts of polymer in 1 mL of a suitable solvent (DMSO, DMF, or acetone) to create 1.73 and 3.46 mg mL^{-1} polymer concentrations. Then the organic phase was added to the aqueous phase (water or PBS 1X or PBS 7X or PVA 0.5-2 wt%). Addition of the organic phase to the aqueous phase was performed either slowly and drop-wise in 15 minutes while stirring at 1000 rpm or fast while stirring at 1500 rpm at 25 °C and 80 °C. Formulations with volatile solvents were stirred overnight for solvent evaporation. Formulations with non-volatile solvent were transferred to the dialysis membrane (MWCO of 3.5 kDa) and were dialyzed against water overnight to remove solvent and excessive PVA.

Table S2. Formulation parameters of empty PSA_{100}-NPs formed by the nanoprecipitation method.

#	Polymer concentration (mg mL^{-1})	Final solution volume (mL)	Solvent	Aqueous phase	Formulation description
P1	3.46	5	Acetone	Water	Drop wise addition of organic phase to aqueous phase (15 min, 10 µl every 10 s) at 1000 rpm, solvent evaporation overnight
P2	3.46	5	Acetone	Water	Drop wise addition of organic phase to aqueous phase (15 min, 10 µl every 10 s) at 1000 rpm, followed by sonication (100% power, 10 min), solvent evaporation overnight
P3	3.46	5	Acetone	PVA 1%	Drop wise addition of organic phase to aqueous phase (15 min, 10 µl every 10 s) at 1000 rpm, solvent evaporation overnight
P4	3.46	5	DMSO	Water	Drop wise addition of organic phase to aqueous phase (15 min, 10 µl every 10 s) at 1000 rpm, centrifuged for 20 min at RT
P5	3.83	5	Acetone	PVA 0.5%	Drop wise addition of organic phase to aqueous phase (15 min, 10 µl every 10 s) at 1000 rpm, solvent evaporation overnight
P6	3.41	5	DMF	PVA 1%	Drop wise addition of organic phase to aqueous phase (15 min, 10 µl every 10 s) at 1000 rpm, dialyzed against Milli-Q water and washed 2X with water
P7	3.69	5	DMF	PVA 1%	Fast addition of organic phase to aqueous phase and stirred the NP suspension for 30s at 1000 rpm, dialyzed against Milli-Q water, and washed 2X with water
P8	4.32	1	DMF	PBS 7X	Dissolved polymer was mixed with 250 µl PBS 7X and then mixed with 800 µl Milli-Q water by gentle pipetting
P9	3.79	5	DMF	PBS 1X	Fast addition of organic phase to aqueous phase and stirred the NP suspension for 30s at 1000 rpm, dialyzed against PBS 1X

P10	3.93	10	DMF	PVA 1%	Fast addition of organic phase to aqueous phase and stirred the NP suspension for 30s at 1000 rpm, dialyzed against Milli-Q water
P11	1.73	5	DMF	PVA 1%	Fast addition of organic phase to aqueous phase and stirred the NP suspension for 30s at 1000 rpm, dialyzed against Milli-Q water and washed 2X with water
P12	3.46	5	DMF	PVA 1%	Fast addition of organic phase to 80°C PVA solution (aqueous phase) and stirred the NP suspension for 30s at 1000 rpm, dialyzed against Milli-Q water and washed 2X with water
P13	3.46	5	DMF	PVA 2%	Fast addition of organic phase to aqueous phase and stirred the NP suspension for 30s at 1000 rpm, dialyzed against Milli-Q water and washed 2X with water
P14	3.46	5	DMF	PVA 1%	Fast addition of organic phase to aqueous phase and stirred the NP suspension for 30s at 1500 rpm, dialyzed against Milli-Q water and washed 2X with water
P15	3.46	5	DMF	PVA 2%	Fast addition of organic phase to aqueous phase and stirred the NP suspension for 30s at 1500 rpm, dialyzed against Milli-Q water and washed 2X with water
P16	3.46	5	DMF	PVA 2%	Fast addition of organic phase to aqueous phase and stirred the NP suspension for 30s at 1500 rpm, sonicated 30s with 100% power dialyzed against Milli-Q water and washed 2X with water

Nile-red loaded NPs by the nanoprecipitation method were synthesized same way as empty NPs formulation P7 (Table S2) and by the addition of 125 mg g^{-1} NR: polymer ratio (0.432 mg NR per 3.46 mg polymer) to the organic phase. The NPs suspension was dialyzed against water to remove solvent and excessive PVA. After dialysis, the non-encapsulated dye was separated by slow-speed centrifugation (2500 rpm) for 20 minutes. Then the supernatant was collected and dried using SpeedVac for 6 hours. Finally, the PSA_{100}-NPs and NR-PSA_{100}-NPs suspensions were either used without any further dilutions or were diluted using previously prepared medium for cell culture, based on the initial polymer concentration and fixed polymer: NR ratio used in the formulation. In this regard, working concentrations of 0.1-1000 µg mL^{-1} (initial polymer concentration) were prepared from PSA_{100}-NPs suspension (Table S3). Further, working concentrations of 0.1, 0.3, and 0.6 mg mL^{-1} (initial polymer concentration) were also prepared from NR-PSA_{100}-NPs suspension (Table S4). The size, PDI, and zeta potential of the NPs were measured by DLS.

Fluorescein-loaded NPs (FL-PSA_{100}-NPs) by the nanoprecipitation method were also synthesized in the same way as empty NP formulation P7 (Table S2) and by the addition of 125 mg g^{-1} Fluorescein: polymer ratio (0.432 mg Fluorescein per 3.46 mg polymer) to the organic phase. The rest of the purification process was carried out as previously described. Finally, the FL-PSA_{100}-NPs suspensions were used without any further dilutions.

Table S3. Dilutions of PSA_{100}-NPs suspensions based on initial polymer concentration.

#	Initial polymer concentration (μg mL^{-1})
PSA_{100}-NPs-a	0.1
PSA_{100}-NPs-b	1
PSA_{100}-NPs-c	10
PSA_{100}-NPs-d	100
PSA_{100}-NPs-e	500
PSA_{100}-NPs-f	600
PSA_{100}-NPs-g	1000

Table S4. Dilutions of NR-PSA_{100}-NPs suspensions based on initial polymer concentration.

#	Initial polymer concentration (mg mL^{-1})
NR-PSA_{100}-NPs-a	0.1
NR-PSA_{100}-NPs-b	0.3
NR-PSA_{100}-NPs-c	0.6
NR-PSA_{100}-NPs-d	3.46

Transmission Electron Microscopy (TEM)

Transmission electron microscopy (TEM) was utilized to examine the morphology of NR-PSA_{100}-NPs using a Thermo Scientific Spectra 300 operated at an accelerating voltage of 300 kV. One drop from NR-PSA_{100}-NPs-d was deposited onto a carbon-coated, pretreated copper grid, and after allowing it to sit for 30 seconds to 5 minutes, the excess solvent was removed with filter paper. The sample was subsequently stained with a 1% uranyl acetate solution for negative contrast and left to air dry before imaging.

Disassembly of PSA_{100}-NPs at various pH values

When utilizing the DLS device, the scattering intensity detected by the APD detector without the laser attenuation filter will be used to generate a calculated parameter in the Zetasizer Nano software named the derived mean count rate. A reduction in this parameter reflects the detection of fewer photons at the detector, which consequently reports the presence of fewer particles when all other parameters are kept constant.[4, 5] To evaluate the acid hydrolysis of empty PSA_{100}-NPs-e, the derived mean count rate of NPs as well as their size and PDI was measured using DLS at varying pH values (pH value of PBS buffer was adjusted to pH=1-7.4 using HCl and NaOH) at different time points while incubating at 37°C.

Evaluation of the biocompatibility of homopolymers

ARPE-19 as well as primary derived Müller cells (P.16 and P.4, respectively) were seeded in transparent 96-well plates (Falcon, Germany) at a density of 15,000 cells per well. The empty PSA_{100}-NPs obtained from nanoprecipitation method were dissolved in Milli-Q water, then diluted with low glucose (1 g L^{-1}) DMEM medium (10% FBS, 1% penicillin G plus streptomycin sulfate) to obtain diluted NPs suspensions based on initial polymer concentrations of 0.1, 1, 10, 100, and 1000 µg mL^{-1} (PSA_{100}-NPs-a to PSA_{100}-NPs-d and PSA_{100}-NPs-g).

The next day after seeding the cells, the original culture medium was removed, and NPs suspensions (100 µL, triplicates) were added to each well. After 24 and 48 hours of incubation, cell viability and cell density were determined using MTS and CV assays, respectively.

In addition, PGA polymers were dissolved in the culture medium (1000 µg mL^{-1}). 100 µL of homopolymer solutions (PGA_{25}, $PGA_{50,}$ and PGA_{100}) was added to each well (triplicates) and incubated for 24 hours. Then, the cell viability and cell density were determined using MTS and CV assays, respectively.

MTS viability assay[6, 7]

After incubation of cells with PSA_{100}-NPs and PGA for 24 hours and 48 hours of incubation for PSA_{100}-NPs, the MTS assay was performed by adding the MTS reagent (20 µL per well, CellTiter 96® AQueous One Solution Reagent, Promega) to the culture medium and incubating for 90 minutes. Then, the absorbance was measured at 490 nm (interference set at 690 nm) using a microplate reader (Infinite 200, TECAN, Maennedorf, Switzerland). The experiments were performed in three sets, with each set including three replicates. For 100% cell viability, the average normalized absorbance of untreated cells as a control was utilized. Finally, the absorbance of each well was divided by the average absorbance of the control, multiplied by 100 to calculate the cell viability % per well.

Crystal violet staining[8]

The cell density per well was investigated using crystal violet staining. After the MTS assay, the medium was removed from each well, and using 4% PFA (Merck, Darmstadt, Germany), cells were fixed for 15 min at room temperature. After fixation, cells were washed using Milli-Q water (three times). Then, the crystal violet solution (Sigma-Aldrich, Taufkirchen, Germany) was used to stain cells, followed by 30 min incubation. Cells were washed multiple times with Milli-Q water to remove excessive staining and dead cells. In the next step, cells were incubated with sodium dodecyl sulfate (1% SDS, Applichem, Darmstadt, Germany) for 1 h. Finally, the

absorbance was recorded at 595 nm (Infinite 200, TECAN, Maennedorf, Switzerland). The experiments were performed in three sets, with each set including three replicates. For 100% cell density, the average normalized absorbance of untreated cells as a control was utilized. Finally, the absorbance of each well was divided by the average absorbance of the control, multiplied by 100 to calculate the cell density % per well.

Cellular uptake of Nile-red loaded PSA_{100}-NPs

To investigate the uptake and internalization of NR-PSA_{100}-NPs-c, ARPE-19 cells (25,000 cells per well, P.44) and primary-derived Müller cells (25,000 cells per well, P.5) were seeded in transparent 24-well plates. After 24 hours, cell media was aspirated and cells were incubated with NR-PSA_{100}-NPs-c (0.6 mg mL^{-1} based on initial polymer concentration in cell media, 500 µL per well), PSA_{100}-NPs-f (0.6 mg mL^{-1} based on initial polymer concentration in cell media, 500 µL per well), free NR (0.432 mg mL^{-1} dissolved in media, undissolved dye was filtered out using PES, sterile, 0.45 µM syringe filter, 500 µL per well) and nothing as control for 24 hours. After incubation, the cell media was aspirated, and the nuclei of cells were stained with Hoechst (Sigma-Aldrich, 1:2,000 dilution with HBSS) for 5 minutes. Then the cells were washed three times with HBSS and imaged using a fluorescence microscope (Axio Observer, Zeiss) using three channels: Bright field, DAPI (Hoechst) and AF555 (NR). The experiment was performed in three sets, with each set including three replicates. Three pictures per well were generated.

Quantitative uptake

For quantitative uptake of NPs in cells, a modified protocol from Shin et al.[9] was employed. In this regard, after incubation time, cells were cultivated and washed with 500 µL twice to remove any free NPs or NR. In this step, the cell suspension was kept on ice to prevent reverse pumping of taken up NPs out. Then, the cells were moved to a flat clear-bottom, black 96-well plate (Falcon, Germany). Since the number of cells in each well may vary and which affects the final fluorescence of the sample, the absorbance of each well was measured at 600 nm (OD600) in addition to its fluorescence (excitation and emission peaks of 485 and 630 nm, respectively). The fluorescence of each well was normalized to its OD600 and used for plotting the bar chart. Finally, an analysis of variance (one-way ANOVA) was conducted using GraphPad Prism 10.

LysoBrite™ Green assay

To further help localize the NR-PSA_{100}-NPs inside cells, LysoBrite™ Green assay (AAT Bioquest) was performed. For this purpose, ARPE-19 cells (10,000 and 15,000 cells per well, P.15) were seeded in transparent 96-well plates. After 24 hours, cell media was aspirated and cells were incubated with no treatment as a control and NR-PSA_{100}-NPs-a, b, and c (in cell media, 100 µL per well) for 24 hours. The next day, the LysoBrite™ working solution (2 µl of LysoBrite™ stock solution was diluted in 1 mL PBS buffer) was prepared. After the incubation time, 100 µL of LysoBrite™ working solution was added to each well and incubated for another 30 minutes to stain the lysosomes. Finally, the nuclei of cells were counterstained with Hoechst (Sigma-Aldrich, 1:2,000 dilution with HBSS) for 5 minutes. Then the cells were washed three times with HBSS and imaged using a fluorescence microscope. The experiment was performed in three sets, with each set including three replicates. Three pictures per well were generated.

Flow cytometry

ARPE-19 cells were seeded in transparent 48-well plates (50,000 cells per well, P.28). After 24 hours, cell media was aspirated and cells were incubated with 200 µL per well of NR-PSA_{100}-NPs-a, -b, and -c prepared at different initial polymer concentrations in cell media (0.1, 0.3, and 0.6 mg/mL respectively), free NR (0.432 mg mL^{-1} dissolved in media, undissolved dye was filtered out using PES, sterile, 0.45 µM syringe filter) and no treatment as control for 24 hours. Three plates were prepared with the mentioned method. One of the plates was incubated at 4 °C for 4 hours, while the other two plates were incubated at 37 °C for 4 and 24 hours. After incubation, the cell media was aspirated, and the cells were trypsinized and transferred to the FACS tubes. Then, 1 mL PBS (1X) was added to each tube, and cells were centrifuged at 300xg and 21°C for 5 minutes. The supernatant was decanted, and the cell nuclei were stained with 4',6-diamidino-2-phenylindole dihydrochloride (DAPI) (100 µL, 10 µg/ml) for 15 minutes. After incubation, 1 mL PBS buffer was added to each tube, and cells were washed as mentioned before. After decanting the supernatant, 50 µL PBS (1X) was added to each tube, and cells were analyzed on a Canto II flow cytometer (Becton Dickinson). Data was analyzed with FlowJo software v10. Cell debris was excluded by gating side-scattered light (SSC) versus forward-scattered light (FSC). To identify singlets, FSC-area was plotted against FSC-height, and events displaying a linear relationship between area and height were selected. Live cells were then gated by plotting DAPI versus FSC and excluding DAPI-positive events. Within this live-cell population, the percentage of Nile Red (NR)-positive cells and the median fluorescence intensity (MFI) of all live cells were quantified in the 488nm laser, Q495 LPXR splitter and ET

530/30 bandpass filter. For each sample, 30,000 gated events were analyzed, and red fluorescence was recorded in all samples. Analysis of variance (one-way ANOVA) was conducted using GraphPad Prism 10.

Studies with porcine retina explants[10]

Porcine eyes provided from a local abattoir were immediately (maximum 3 hours after being sacrificed) transported to the lab while maintaining eyes at 4 °C. Within 1h of arrival, eyes were cleaned using scissors and sanitized by submerging in 70% ethanol for 5 minutes, followed by two washing steps using PBS and settled to reach room temperature. The eyes were placed in a petri dish held steady with one hand, and each eye was opened using a scalpel under sterile conditions, followed by removal of lens, cornea, and vitreous humor, and cut into a clover shape. Then an 8 mm punch was used to cut out the retinal explants. By the means of two small spatulas and few drops of medium, retina punches were separated from the rest of the eye and were transferred to inserts in 12-well plates with retina cultivation medium (50 mL of retina medium consist of 48 mL Neurobasal media, 2 mL N2, 1 mL B27, 0.5 mL P/S, 0.05 mL Gentamycin, 0.5 mL CNTF and 0.5 mL BDNF). Then, either no treatment, NR-PSA$_{100}$-NPs-c (100 µL, in retina medium) or free NR (100 µL, 0.432 mg dissolved in 1 mL retina media filtered through 0.45 µm PES filter) suspensions were poured on retina explants as treatment (n = 3 for each condition) and incubated for 24 hours at 37 °C and 5% CO_2. After incubation, retina explants were washed with PBS (three times), fixed with PFA 4% (10 min), and counterstained with NucBlue (2 drops of NucBlue Live Cell Stain ReadyProbes diluted in 1 mL PBS, 5 min incubation at RT). Explants were embedded in Tissue-Tek O.C.T. (Sakura Finetek) and subsequently frozen in liquid nitrogen. Thereafter, slides of cryosections were prepared and imaged.

Studies with porcine retina in the eye cup

In line with the process used for retinal explants, with some additional modifications applied, porcine eyes were cleaned and sanitized. After opening the eye, the lens and vitreous humor were removed while keeping the retina intact in the optic cup. Afterwards, no treatment or NR-PSA$_{100}$-NPs-d (150 µL, in retina medium) or free NR (150 µL, 0.432 mg dissolved in 1 mL retina media filtered through 0.45 µm PES filter) suspensions were poured on the retina laying in the eye cup (n = 3 for each condition) and incubated for 24 hours at 37 °C and 5% CO_2. After incubation, eye cups including retina were washed with PBS (three times), fixed with PFA 4% (10 min), and counterstained with NucBlue (5 min). Then the eye cups were embedded in

Tissue-Tek O.C.T. (Sakura Finetek) and subsequently frozen in liquid nitrogen. Thereafter, slides of cryosections were prepared and imaged.

Studies with porcine retina in the complete eye by intravitreal injection

As described previously, porcine eyes from a local abattoir were received (maximum 3 hours after being sacrificed) and transported to the lab while maintaining the eyes at 4 °C fridge. Within 1h of arrival, eyes were cleaned using scissors and sanitized by submerging in 70% ethanol for 5 minutes, followed by two washing steps using PBS and settled to reach room temperature. For NR-PSA_{100}-NPs administration, the eyes were placed on the bottom of a petri dish and fixated with one hand. Afterwards, the *pars plana* was penetrated approximately 4 mm posterior to the limbus of the eye using a needle of 30 gauge (APROTEK GmbH, Germany). Then, 150 µL of NR-PSA_{100}-NPs-d suspension (in PBS) was injected into the vitreous close to the retina at the back side of the eye. Porcine eyes were incubated for 6 h in PBS. For microscopy, the porcine eyes (n = 3) were embedded in Tissue-Tek and subsequently frozen in liquid nitrogen and stored at -20 °C freezer.

Fluorescent Microscopy and Imaging[11]

Frozen sections of porcine eyes were cut (14 µm, longitudinally) on a cryostat (Leica CM 1900), thaw-mounted onto glass slides (Superfrost plus, R. Langenbrinck Labor- & Medizintechnik), and stored at -20 °C for later use. For visualization each section was mounted with Fluorsave mounting medium (unless otherwise specified) topped with cover slips (R. Langenbrinck Labor- & Medizintechnik). Finally, the prepared sections were imaged using a fluorescence microscope (KEYENCE, BZ-X800-Series). The nuclear stain was imaged *via* a DAPI filter whereas the fluorescent NR dye encapsulated in the NPs was captured utilizing an AF555 filter (wavelength of excitation/emission: 553/637 nm). The obtained images were merged to visualize the location of the NR within cells with respect to the nuclei of the cells. Finally, the resulting images obtained from eyes incubated with NR-PSA_{100}-NPs or free NR were compared to the control group.

2. NMR spectroscopy

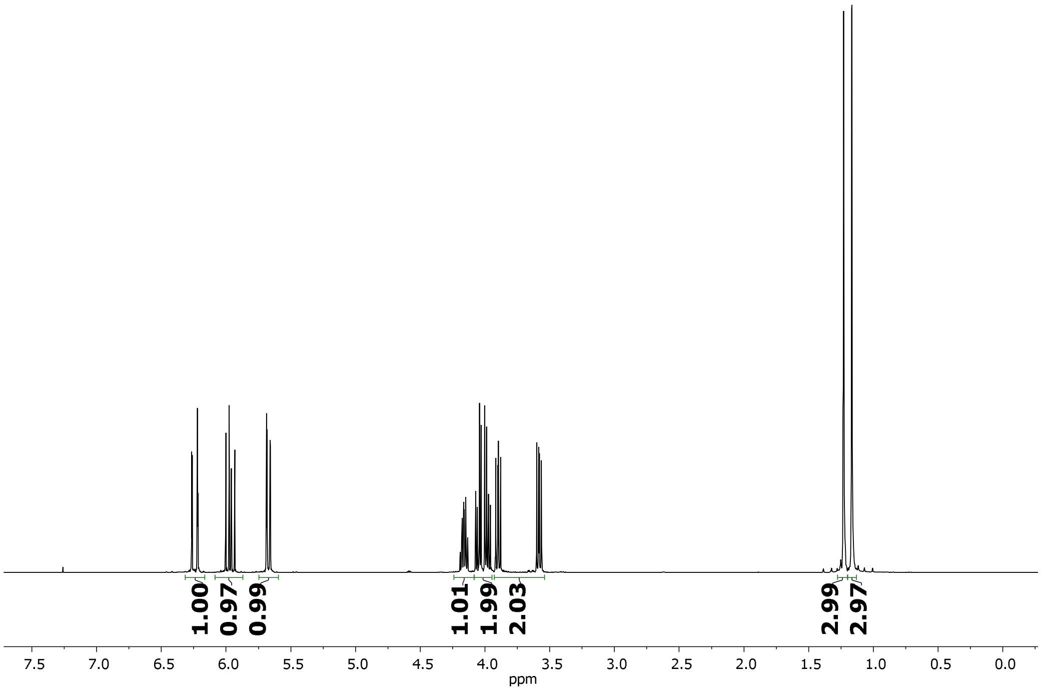

Figure S1. ^{1}H-NMR spectrum of solketal acrylate, 400 MHz, $CDCl_3$, δ (ppm) = 6.24 (dd, J = 17.3, 1.5 Hz, 1H), 5.97 (dd, J = 17.3, 10.4 Hz, 1H), 5.67 (dd, J = 10.4, 1.5 Hz, 1H), 4.16 (qd, J = 6.0, 4.7 Hz, 1H), 4.10 – 3.94 (m, 2H), 3.94 – 3.53 (m, 2H), 1.23 (d, J = 0.9 Hz, 3H), 1.20 – 1.13 (m, 3H).

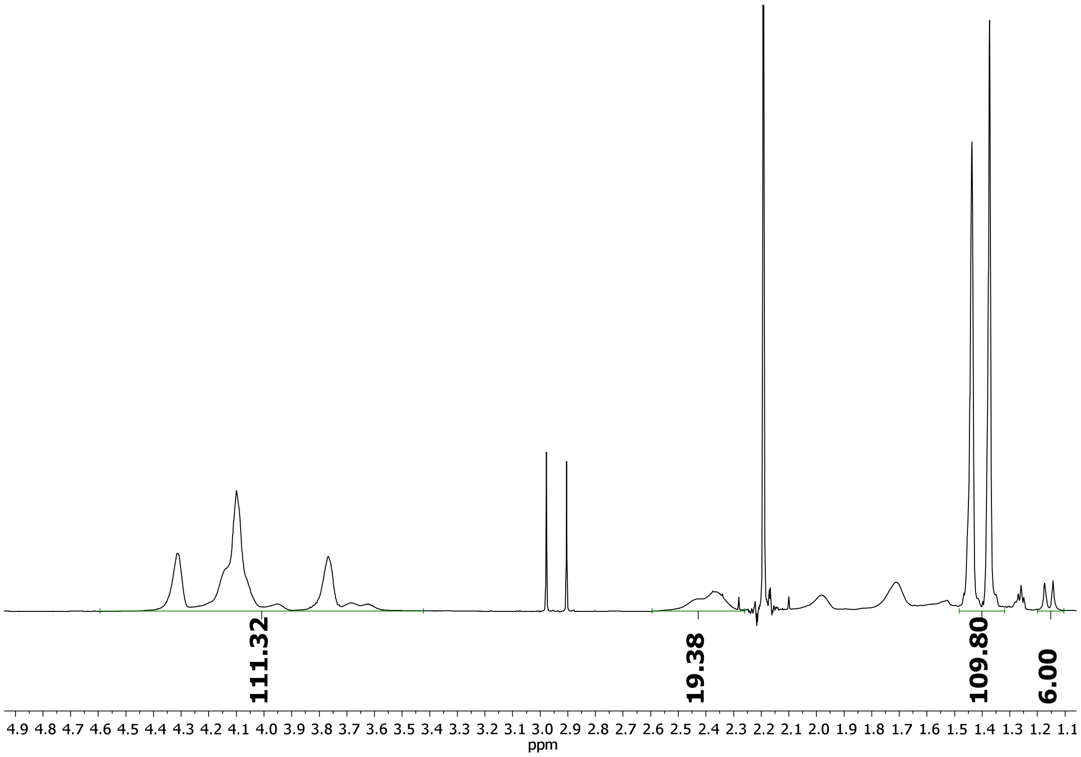

Figure S2. ^{1}H-NMR spectrum of PSA_{25}, 700 MHz, $CDCl_3$, δ (ppm, repeating unit) = 4.29 – 3.59 (m, 5H), 2.31 (m, 1H), 1.75 (m, 2H), 1.35 (s, 3H), 1.28 (s, 3H).

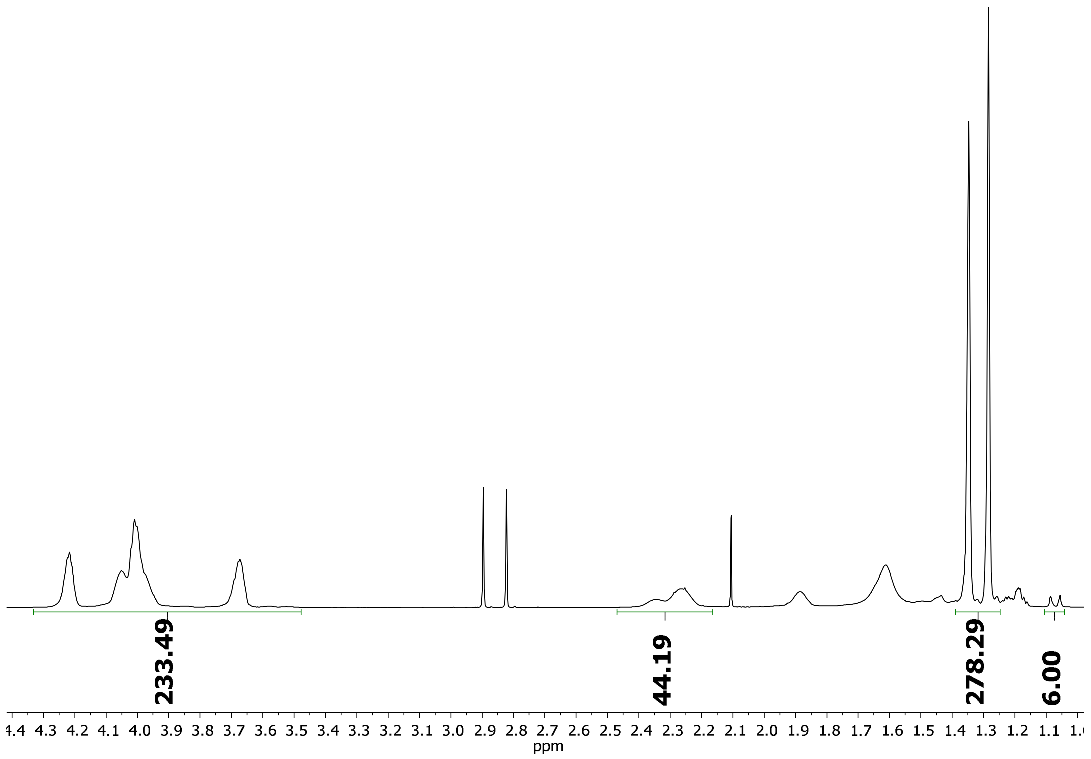

Figure S3. ^{1}H-NMR spectrum of PSA_{50}, 700 MHz, $CDCl_3$.

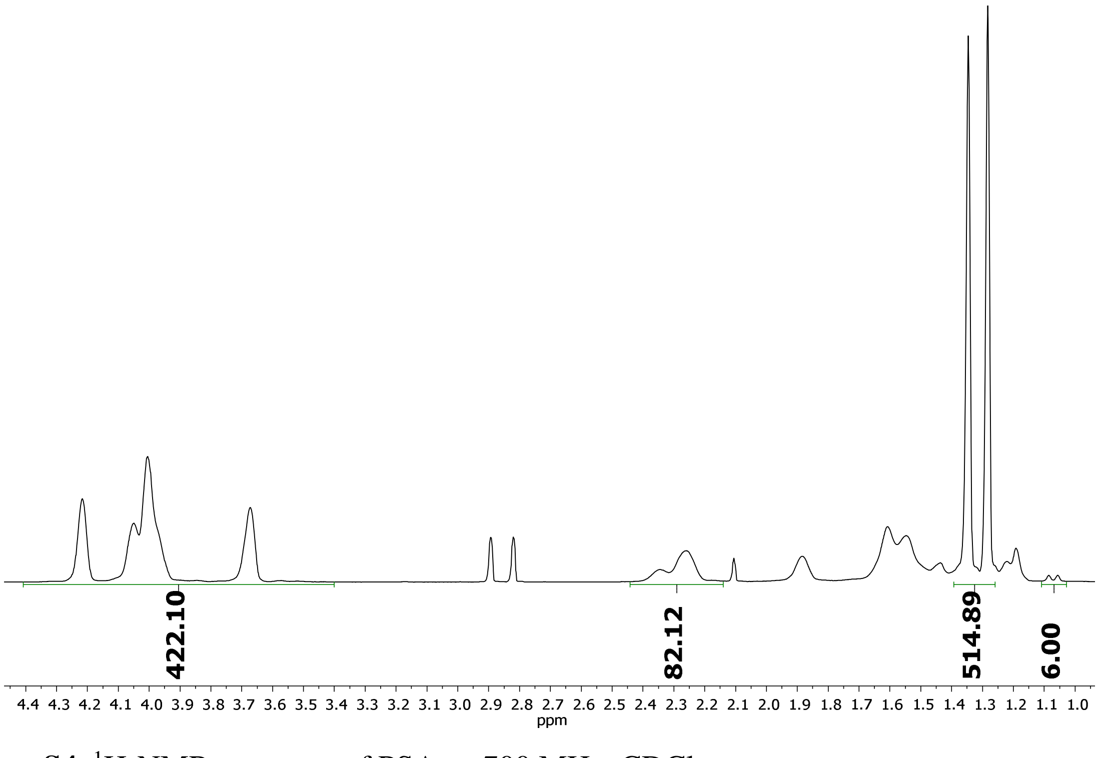

Figure S4. ^{1}H-NMR spectrum of PSA_{100}, 700 MHz, $CDCl_3$.

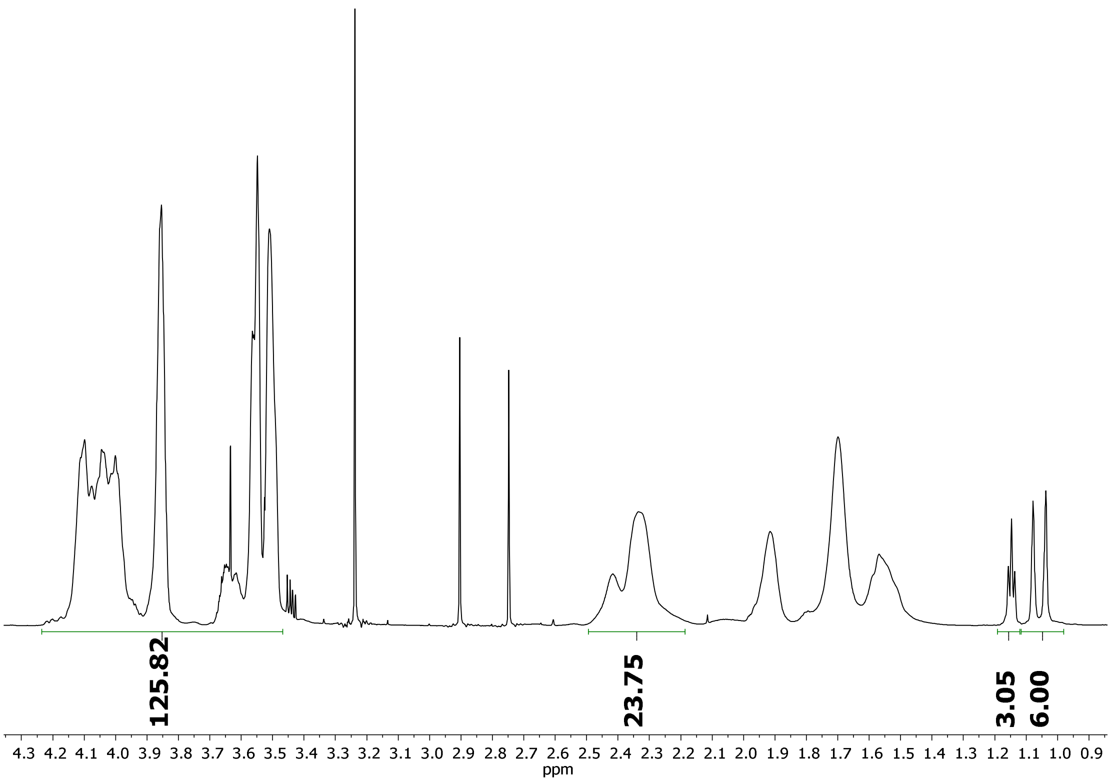

Figure S5. ^{1}H-NMR spectra of PGA_{25}, 700 MHz, $CDCl_3$.

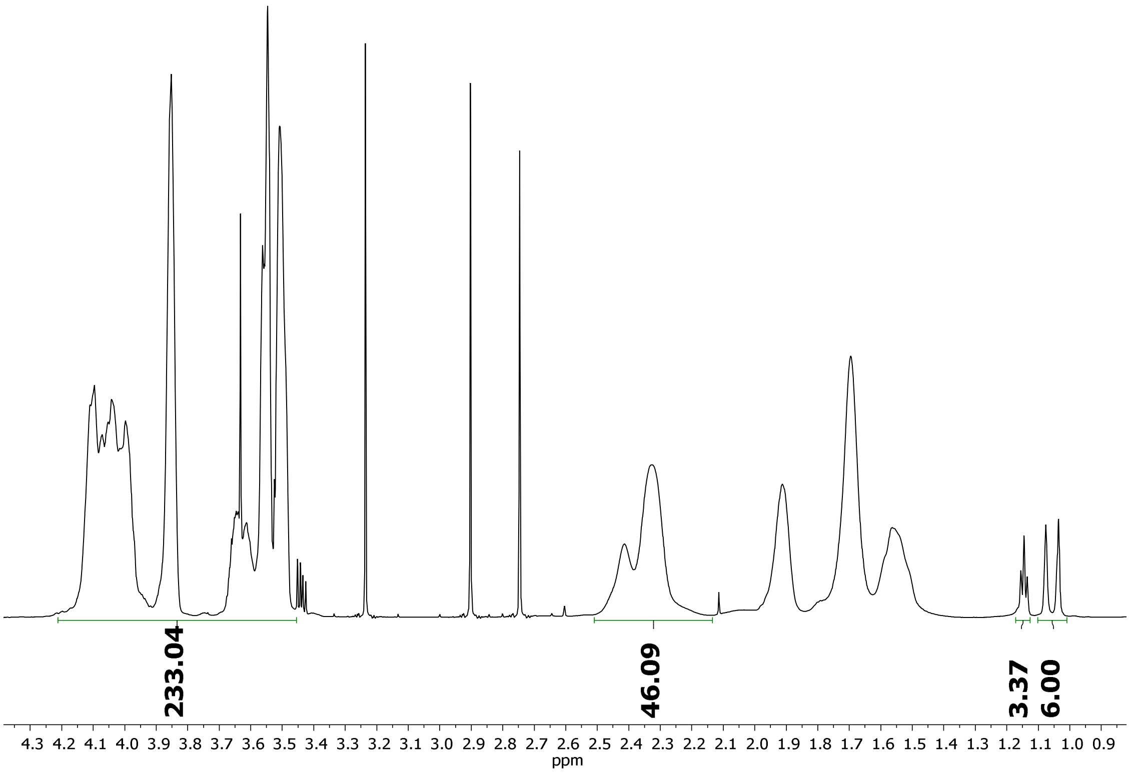

Figure S6. ^{1}H-NMR spectra of PGA_{50}, 700 MHz, $CDCl_3$.

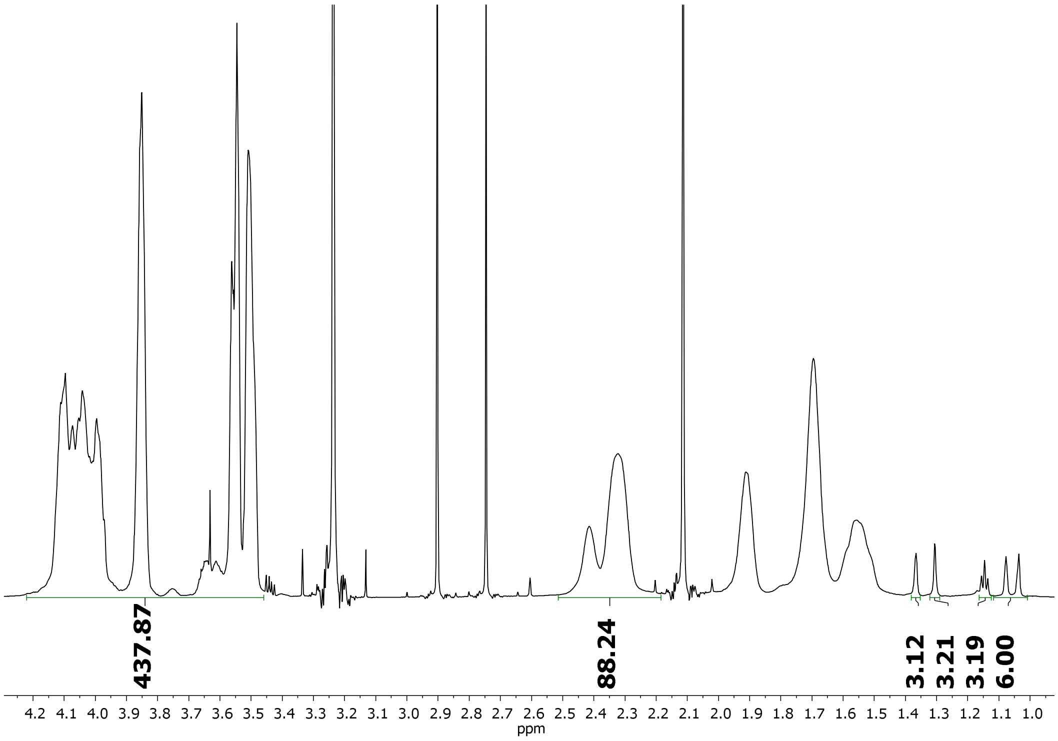

Figure S7. ^{1}H-NMR spectra of $PGA_{100,}$ 700 MHz, $CDCl_3$. One repeating unit per polymer chain is still protected.

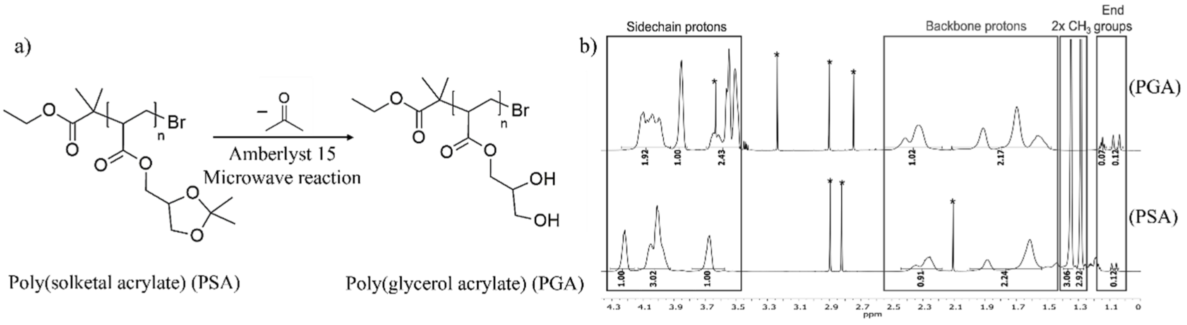

Figure S8. a) Deprotection of PGA using Amberlyst 15 in a microwave reaction. b) ^{1}H-NMR spectra of PSA_{50} and PGA_{50} (* = residual solvent).

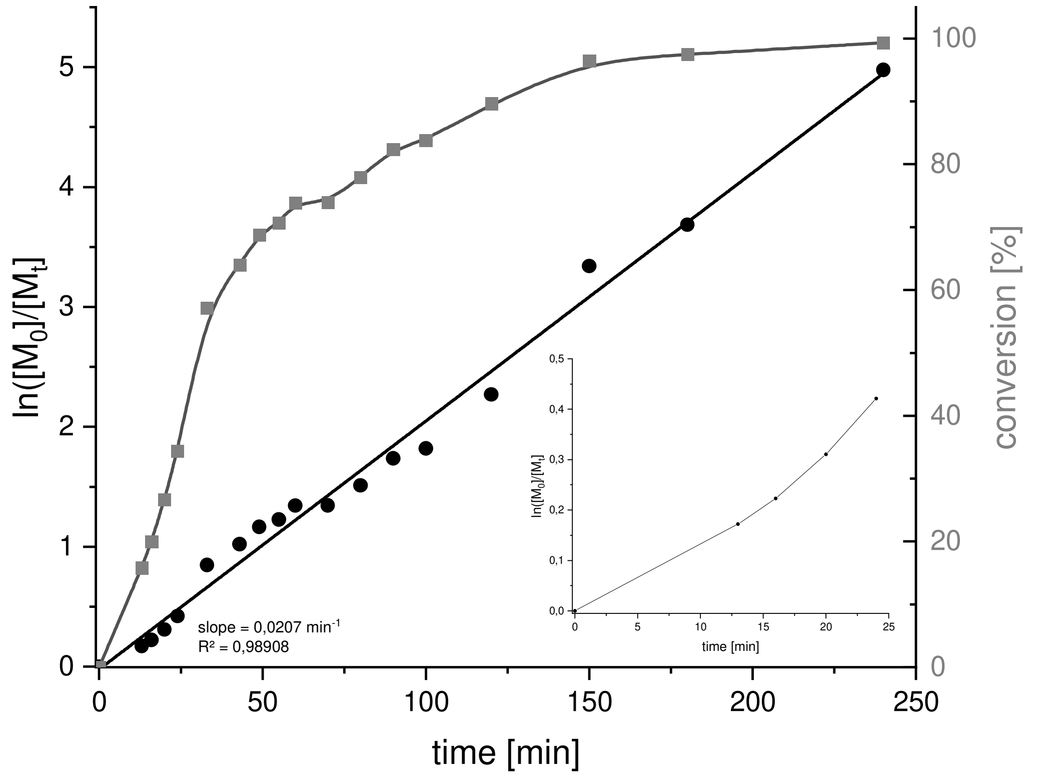

Figure S9. Linear first-order kinetic plot for the polymerization of PSA_{50} at room temperature and zoom in into the initial period of polymerization (t = 0-25 min).

3. IR Spectroscopy

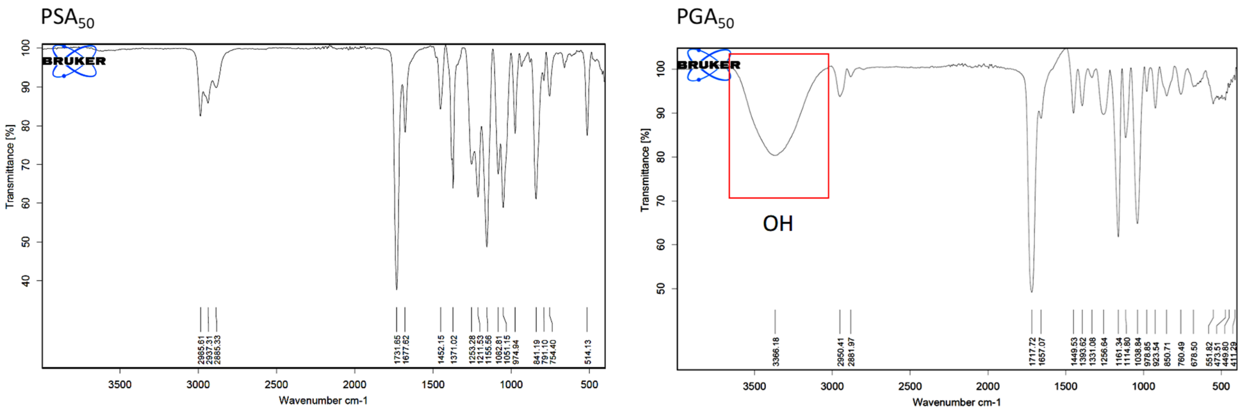

Figure S10. FTIR spectra of PSA_{50} and PGA_{50}.

4. DLS results

Table S5. Size and PDI of empty PSA_{100}-NPs formed by the single emulsion solvent evaporation method determined by DLS. All formulations yielded stable NPs.

Formulation	Size (nm) ± SD	PDI ± SD	Comments	Observed Effect	Ref.
**Variation of Polymer Concentration**					
SE9 (0.69 mg mL^{-1})	134 ± 1.84	0.09 ± 0.02	Stable NPs	Moderate size with low PDI	–
SE4 (2.5 mg mL^{-1})	140 ± 0.96	0.03 ± 0.01	Stable NPs	Increased polymer concentration slightly increased size	[12]
SE10 (0.4 mg mL^{-1})	174 ± 29.6	0.33 ± 0.13	Stable NPs	Larger NPs likely due to insufficient PSA	–
**Variation of PVA Concentration**					
SE11 (0.5 wt%)	147 ± 1.28	0.14 ± 0.04	Stable NPs	Lower PVA concentration yielded smaller NPs	
SE10 (1 wt%)	174 ± 29.6	0.33 ± 0.13	Stable NPs	Higher PVA concentration increased size and PDI	[13-15]
SE5 (1 wt%)	134 ± 1.23	0.09 ± 0.02	Stable NPs	Lower size vs. 2 wt% PVA	–
SE8 (2 wt%)	142 ± 0.44	0.13 ± 0.04	Stable NPs	Larger NPs due to viscosity increase in aqueous phase	[13-15]
**Variation of PVA Molecular Weight**					
SE5 (13k–23k)	134 ± 1.23	0.09 ± 0.02	Stable NPs	Low Mw PVA led to smaller NPs	–
SE7 (146k–186k)	571 ± 25.5	0.37 ± 0.06	Stable NPs	Significantly increased size with high Mw PVA	[16]
**Variation of Sonication Power**					
SE1 (50%)	214 ± 3.29	0.05 ± 0.05	Stable NPs	Lower power resulted in larger NPs	[17]
SE2 (80%)	204 ± 0.78	0.04 ± 0.01	Stable NPs	Increased power reduced size slightly	[17]
SE5 (80%)	134 ± 1.23	0.09 ± 0.02	Stable NPs	Reference for sonication power comparison (smallest size)	–
SE6 (100%)	138 ± 0.45	0.07 ± 0.01	Stable NPs	Slight size increase vs SE5, likely due to threshold effect	–
**Variation of Sonication Duration**					
SE3 (10 min)	150 ± 2.02	0.08 ± 0.01	Stable NPs	Shorter sonication yielded slightly larger NPs	[18]
SE5 (20 min)	134 ± 1.23	0.09 ± 0.02	Stable NPs	Longer sonication produced smaller NPs	[18]
**Variation of Aqueous Phase Volume**					
SE12 (2.5 mL)	148 ± 0.62	0.06 ± 0.02	Stable NPs	Smallest volume resulted in smaller NPs	[19]
SE10 (5 mL)	174 ± 29.6	0.33 ± 0.13	Stable NPs	Increased volume led to larger size and higher PDI compared to SE12	[19]
SE4 (5 mL)	140 ± 0.96	0.03 ± 0.01	Stable NPs	Control reference (smallest size)	–
SE3 (10 mL)	150 ± 2.02	0.08 ± 0.01	Stable NPs	Further increase in volume led to moderately larger NPs compared to SE4	[19]

Table S6. Size and PDI of empty PSA_{100}-NPs formed by the nanoprecipitation method determined by DLS.

Formulation	Size (nm) ± SD	PDI ± SD	Comments	Observed Effect	Ref.
			**Effect of stabilizer & sonication (acetone solvent)**		
P1 (Water, no sonication)	154 ± 2.48	0.05 ± 0.02	Unstable NPs	No stabilizer used, zeta potential -21.08 mV	–
P2 (Water + sonication)	303 ± 119	0.33 ± 0.09	Unstable NPs	Sonication after mixing increased size & PDI; polymer separation observed	–
P3 (1% PVA)	204 ± 1.29	0.07 ± 0.02	Stable NPs with PVA coating	Less negative zeta potential (-14.08 mV) compared to uncoated P1 formulation	[20, 21]
P5 (0.5% PVA)	208 ± 1.84	0.04 ± 0.03	Stable NPs	Less PVA concentration yielded slightly larger NPs	–
			**Effect of mixing speed & addition rate**		
P6 (Slow addition, 1000 rpm mixing rate)	172 ± 0.79	0.11 ± 0.03	Stable NPs	Intermediate size, reference for mixing speed and addition rate comparison	[22-25]
P7 (Fast addition, 1000 rpm mixing rate)	171 ± 1.38	0.06 ± 0.00	Stable NPs	Faster addition reduced size slightly, slight increase in PDI	[22]
P14 (Fast addition, 1500 rpm mixing rate)	165 ± 2.90	0.16 ± 0.03	Stable NPs	Mixing at 1500 rpm didn't reduce size further compared to 1000 rpm	[26]
			**Effect of temperature**		
P7 (25 °C)	171 ± 1.38	0.06 ± 0.00	Stable NPs	Reference for temperature comparison	–
P12 (80 °C)	145 ± 1.21	0.03 ± 0.02	Smaller size and reduced PDI	Increased temperature reduced size and PDI significantly	[27]
			**Effect of PVA concentration (DMF solvent)**		
P7 (1% PVA)	171 ± 1.38	0.06 ± 0.00	Stable NPs	Reference for PVA concentration comparison	–
P13 (2% PVA)	144 ± 0.62	0.05 ± 0.03	Smaller and stable NPs	Higher PVA (2%) with mixing at 1000 rpm produced smallest NPs	[28, 29]
P15 (2% PVA, 1500 rpm mixing rate)	148 ± 1.87	0.11 ± 0.04	Stable NPs	Extra sonication did not change size, slight PDI reduction	–
P16 (2% PVA + sonication)	148 ± 2.30	0.08 ± 0.05	Stable NPs	Similar to P15 with slightly lower PDI after sonication	[30, 31]
			**Other conditions**		
P4 (PBS 1X)	–	–	No NPs formed	–	–
P8 (PBS 7X)	–	–	Aggregation observed	–	–
P9 (PBS 7X)	–	–	Aggregation observed	–	–
P10	–	–	Aggregation observed	–	–
P11 (Low polymer conc.)	763 ± 258	0.65 ± 0.23	Unstable, large aggregates	Low polymer concentration failed to form stable NPs	–

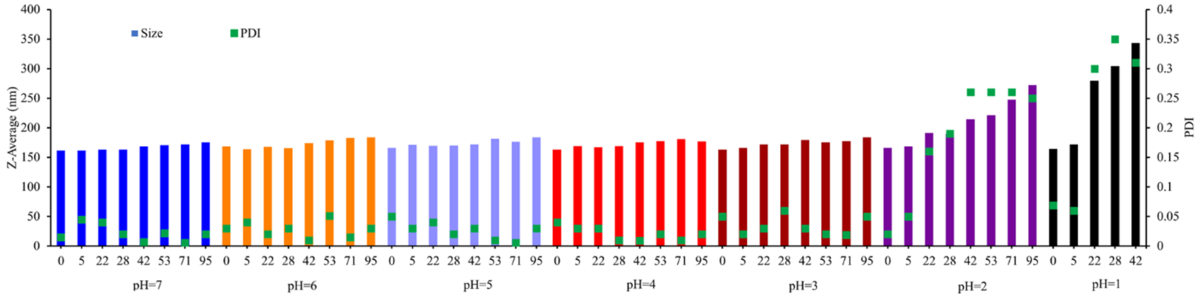

Figure S11. Z-average and PDI of PSA$_{100}$-NPs after incubation at pH ranging 1-7 for up to 95 hours.

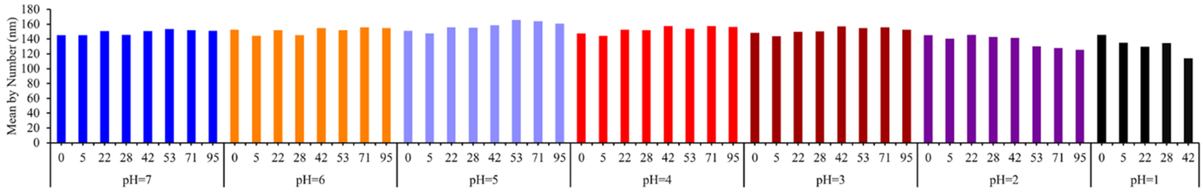

Figure S12. Mean by number of PSA$_{100}$-NPs after incubation at pH ranging 1-7 for up to 95 hours.

5. Transmission Electron Microscopy (TEM)

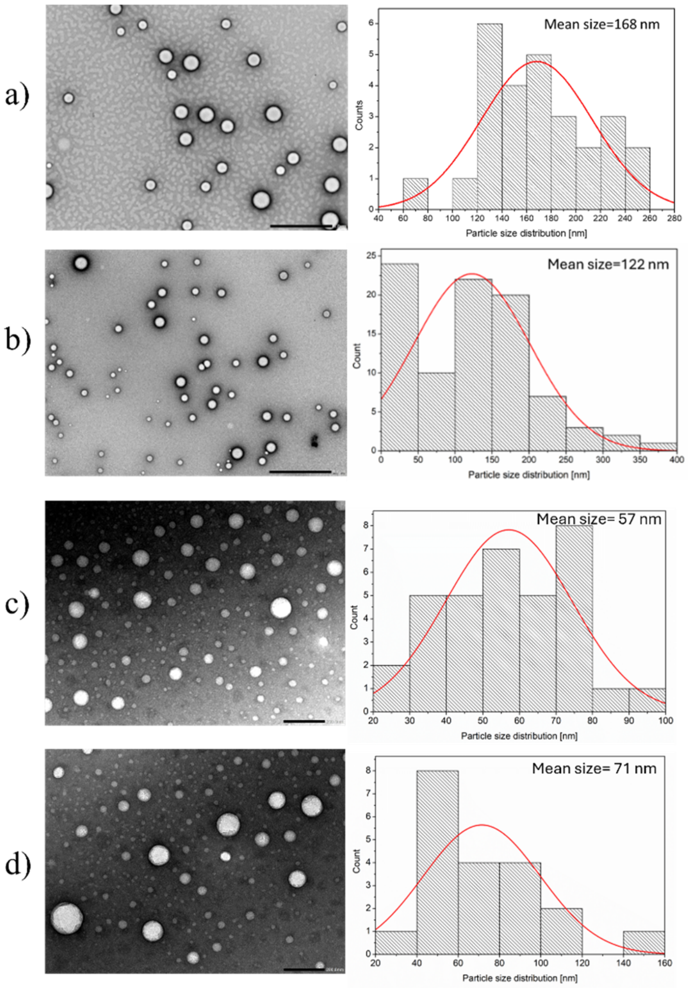

Figure S13. TEM images of spherical nanoparticles analysed using ImageJ. a, b) NR-PSA$_{100}$ NPs with scale bars of 1 µm and 2 µm, respectively. c, d) Empty PSA$_{100}$ NPs with scale bars of 200 nm.

5. Biocompatibility assays

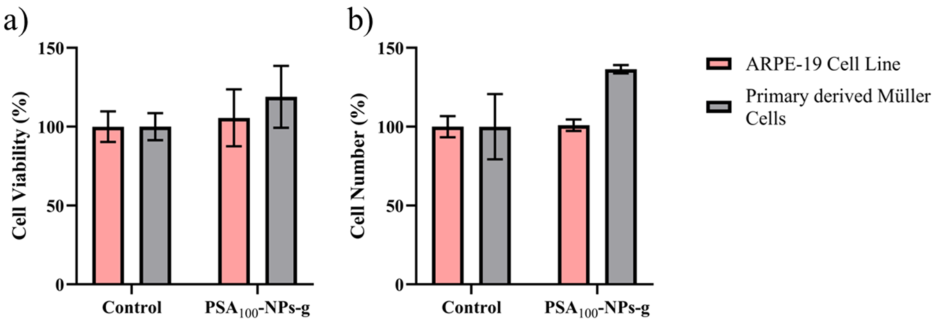

Figure S14. MTS and CV assays were conducted using PSA_{100}-NPs (1000 µg mL^{-1} based on initial polymer concentration) with the ARPE-19 cell line and primary-derived Müller cells. The culture duration for each assay was 48 hours (n = 3 ± SD).

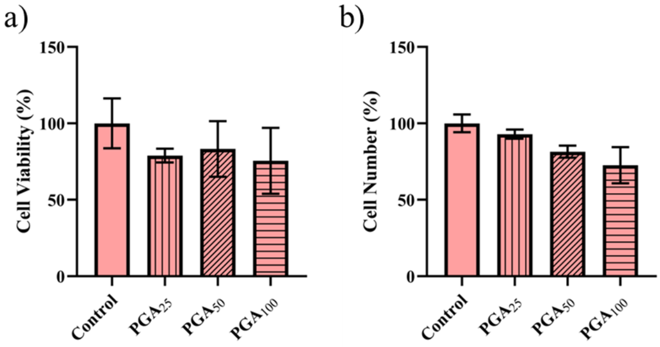

Figure S15. MTS (a) and CV (b) assays were conducted utilizing 1000 µg mL^{-1} concentration of PGA_{25}, PGA_{50}, and PGA_{100} with the ARPE-19 cell line. The culture duration for each assay was 24 h (n = 3 ± SD).

6. Cellular uptake studies

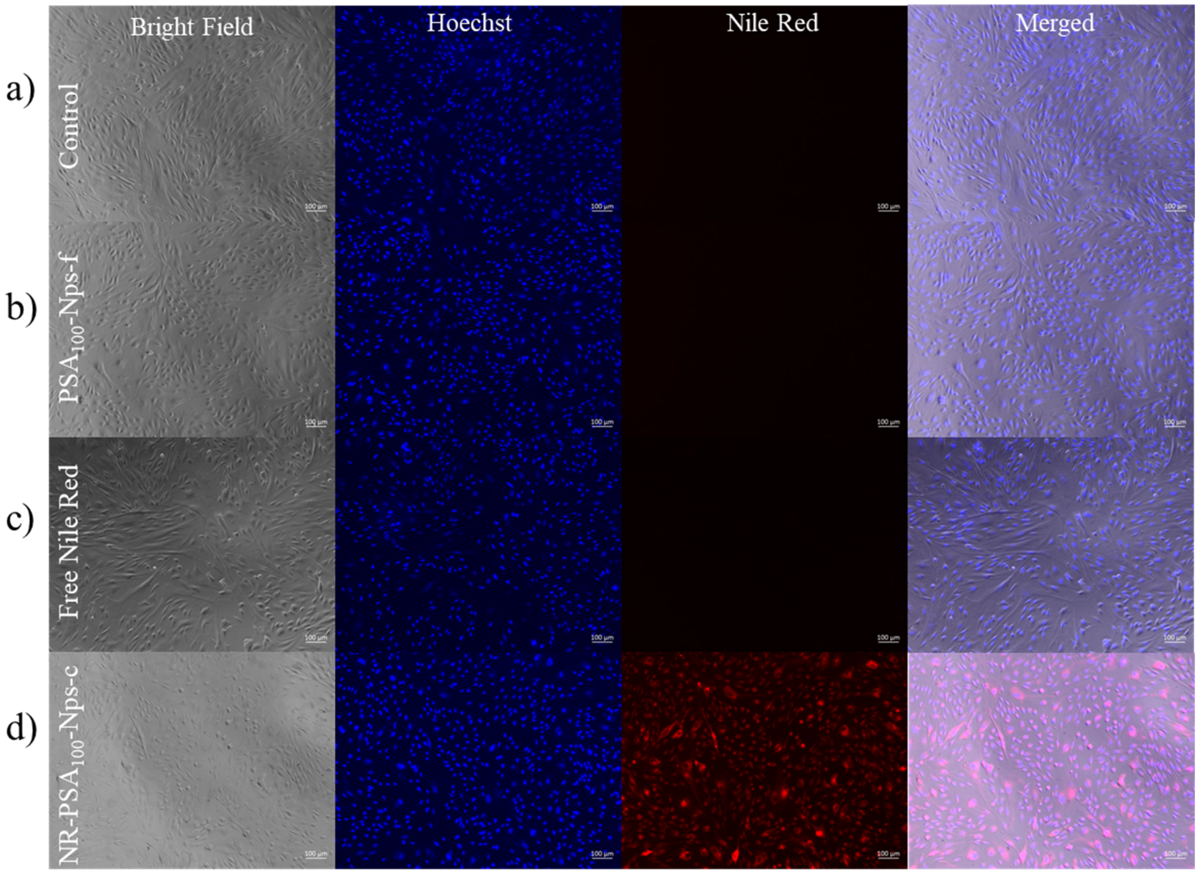

Figure S16. Fluorescence images (100X magnification) of primary-derived Müller cells incubated with a) no treatment as control, b) empty PSA_{100}-NPs-f, c) free NR, d) NR-PSA_{100}-NPs-c for 24 hours. Cells were imaged through bright field, DAPI channel (for Hoechst staining), and AF555 channel (for NR fluorescence). Merged images were generated by the software. A scale bar of 100 μm was included for reference.

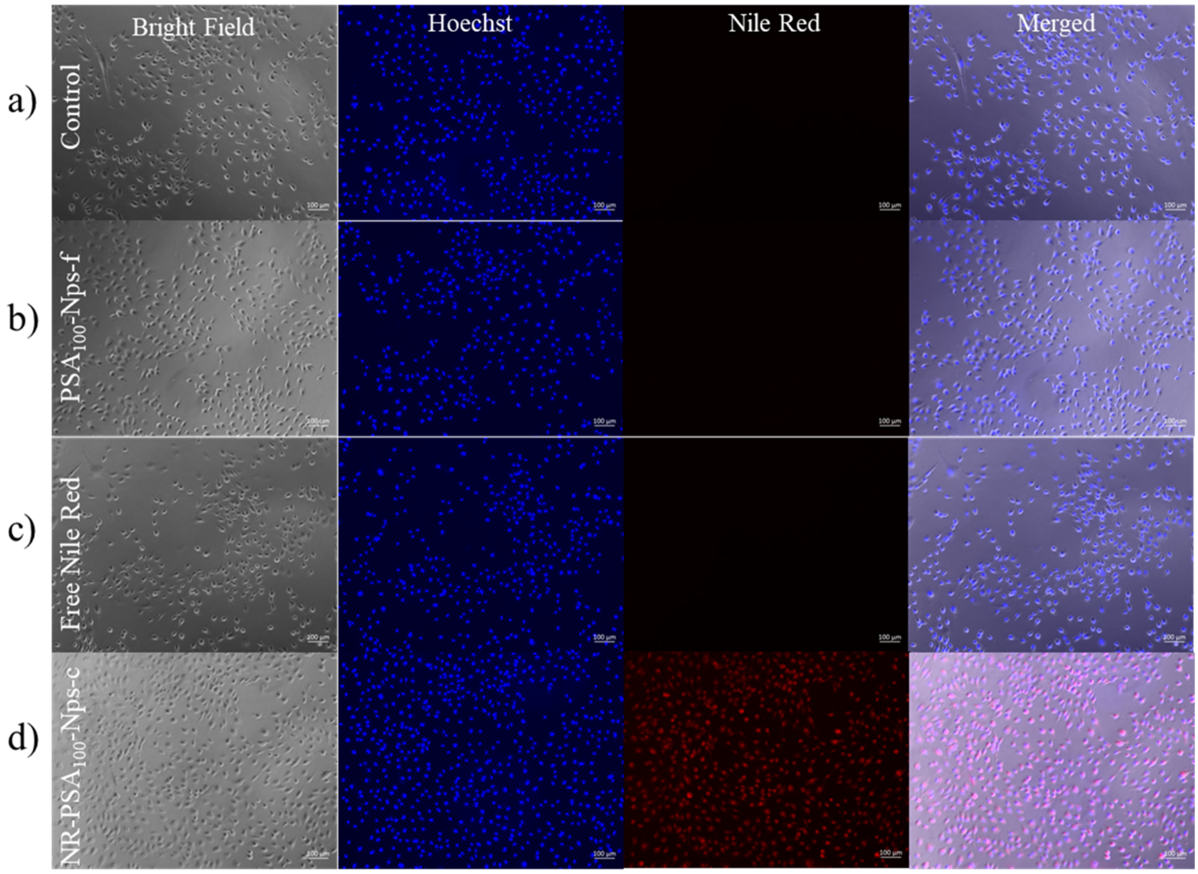

Figure S17. Fluorescence images (100X magnification) of ARPE-19 cells incubated with a) no treatment as control, b) empty PSA_{100}-NPs-f, c) free NR, d) NR-PSA_{100}-NPs-c, for 24 hours. Further, cells were imaged through bright field, DAPI channel (for Hoechst staining), and AF555 channel (for NR fluorescence). Merged images were generated by the software. A scale bar of 100 µm was included for reference.

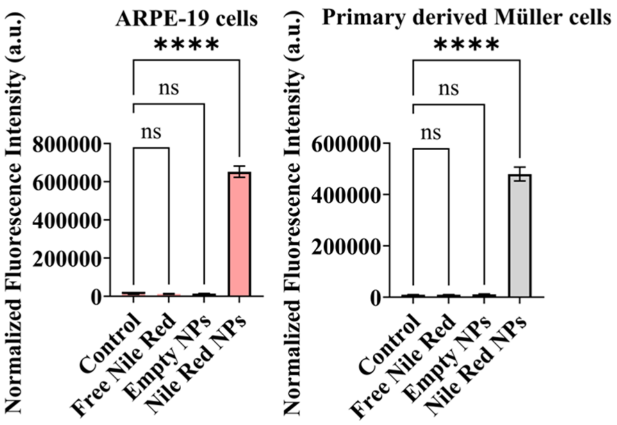

Figure S18. Quantitative analysis of fluorescence imaging of ARPE-19 and primary-derived Müller cells incubated for 24 hours with NR-PSA_{100}-NPs, free NR, empty PSA_{100}-NPs, and an untreated control. The significance levels are indicated in the plots as: * for $p \leq 0.05$, ** for $p \leq 0.01$, *** for $p \leq 0.001$, and **** for $p \leq 0.0001$.

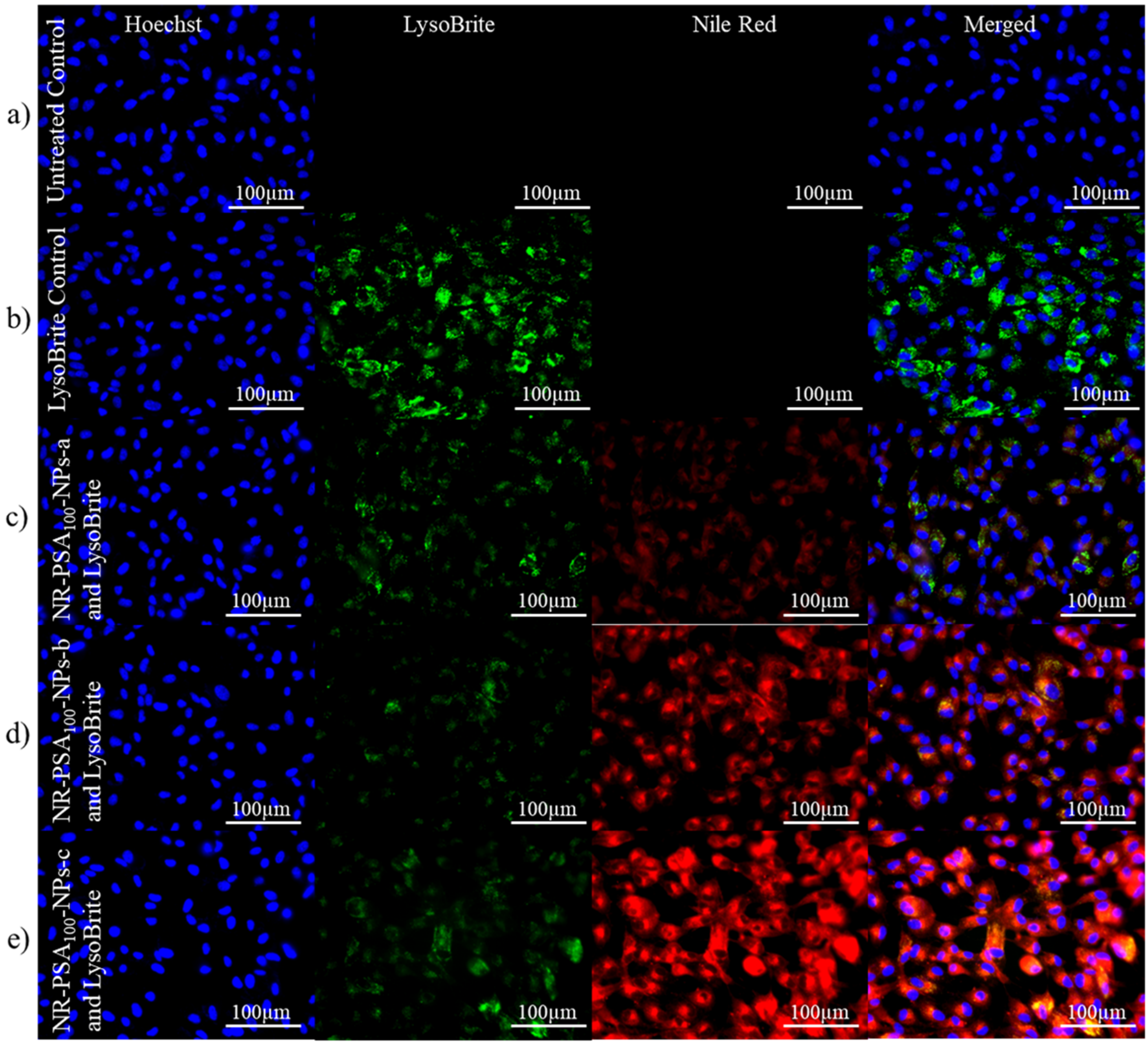

Figure S19. ARPE-19 cells incubated with a) no treatment as control; b) LysoBrite only, c) NR-PSA$_{100}$-NPs-a, d) NR-PSA$_{100}$-NPs-b, and e) NR-PSA$_{100}$-NPs-c for 24 hours. After incubation, all NPs treated groups, as well as one of the control groups, were stained with LysoBrite Green for 30 minutes. In addition, all groups were counterstained with Hoechst to stain the nuclei of cells. Fluorescence images (400X magnification) using blue (Hoechst), green (LysoBrite Green), and red (NR-PSA$_{100}$-NPs) channels were taken from samples, and a scale bar of 100 µm was included for reference.

7. Flow cytometry

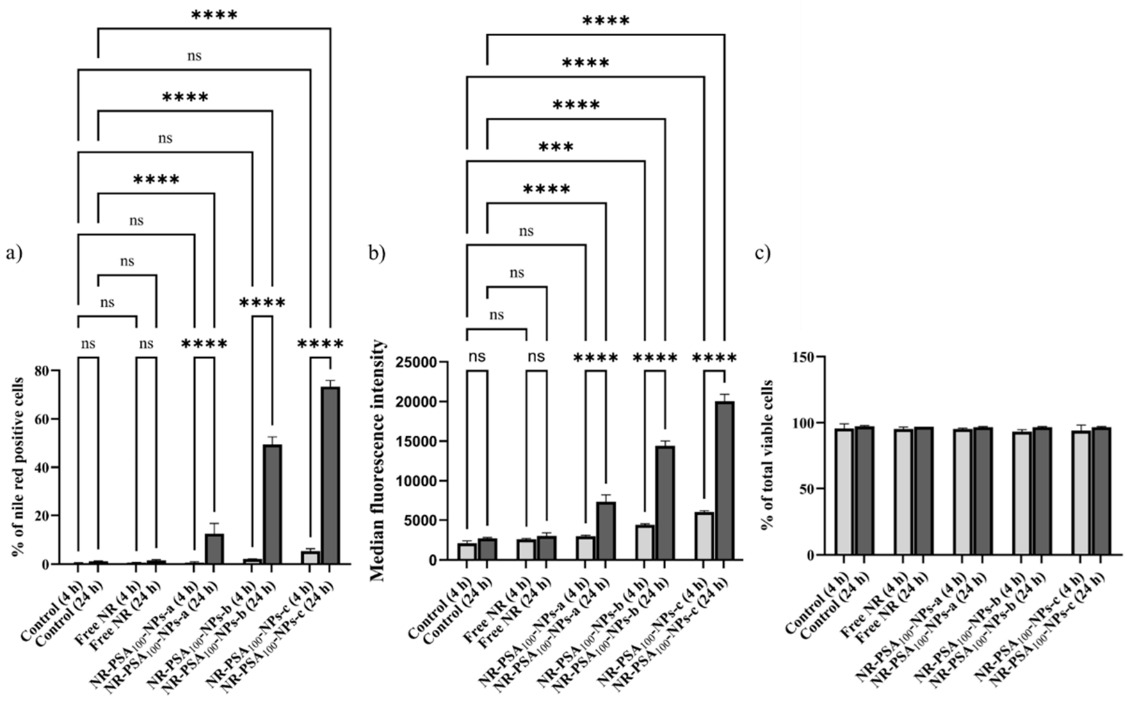

Figure S20. The bar graphs show summary data of flow cytometry analysis from treatments with control, free NR, and NR-PAS$_{100}$-NPs after 24 h incubation at 37°C. a) percentage of NR-positive cells, b) the median NR fluorescence intensity, and c) the percentage of total viable cells. The significance levels are indicated in the plots as: * for p ≤ 0.05, ** for p ≤ 0.01, *** for p ≤ 0.001, and **** for p ≤ 0.0001.

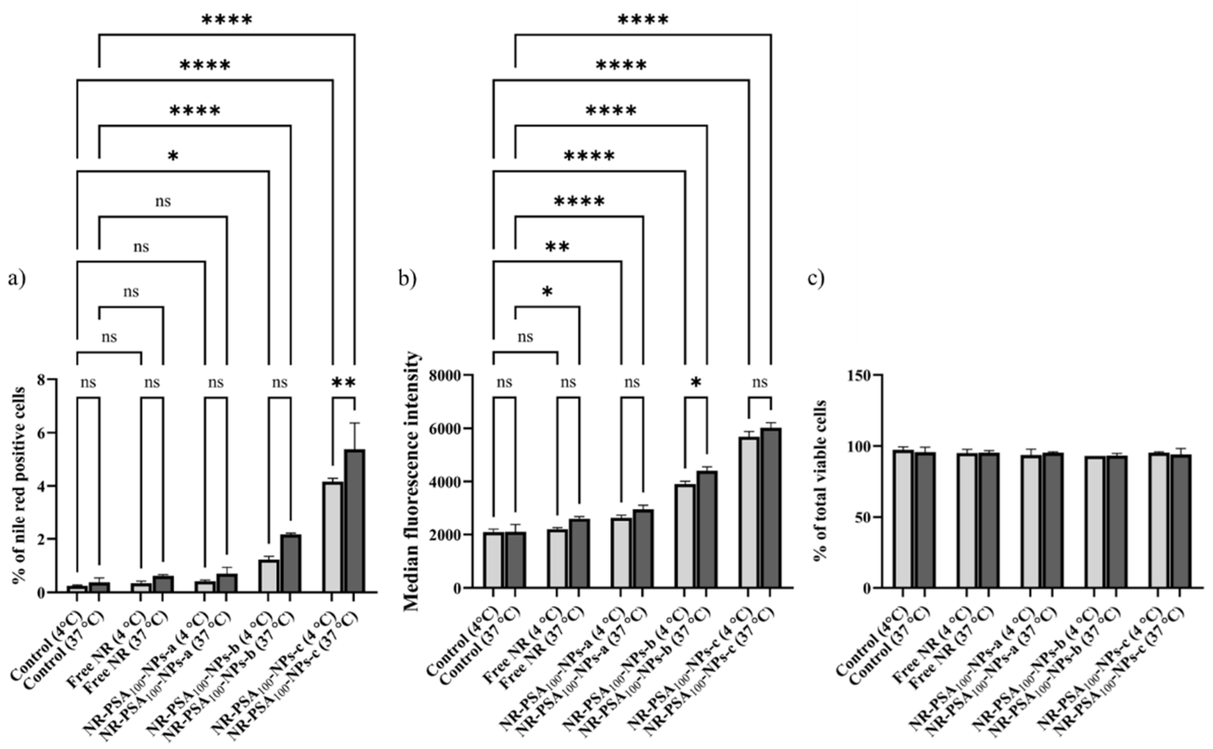

Figure S21. The bar graphs show summary data of flow cytometry analysis from treatments with control, free NR, and NR-PAS$_{100}$-NPs after 4 h incubation at 4 °C vs 37 °C. a) percentage of NR positive cells, b) the median NR fluorescence intensity, and c) the percentage of total viable cells. The significance levels are indicated in the plots as: * for $p \leq 0.05$, ** for $p \leq 0.01$, *** for $p \leq 0.001$, and **** for $p \leq 0.0001$.

8. Ex vivo studies

a)

b)

c)

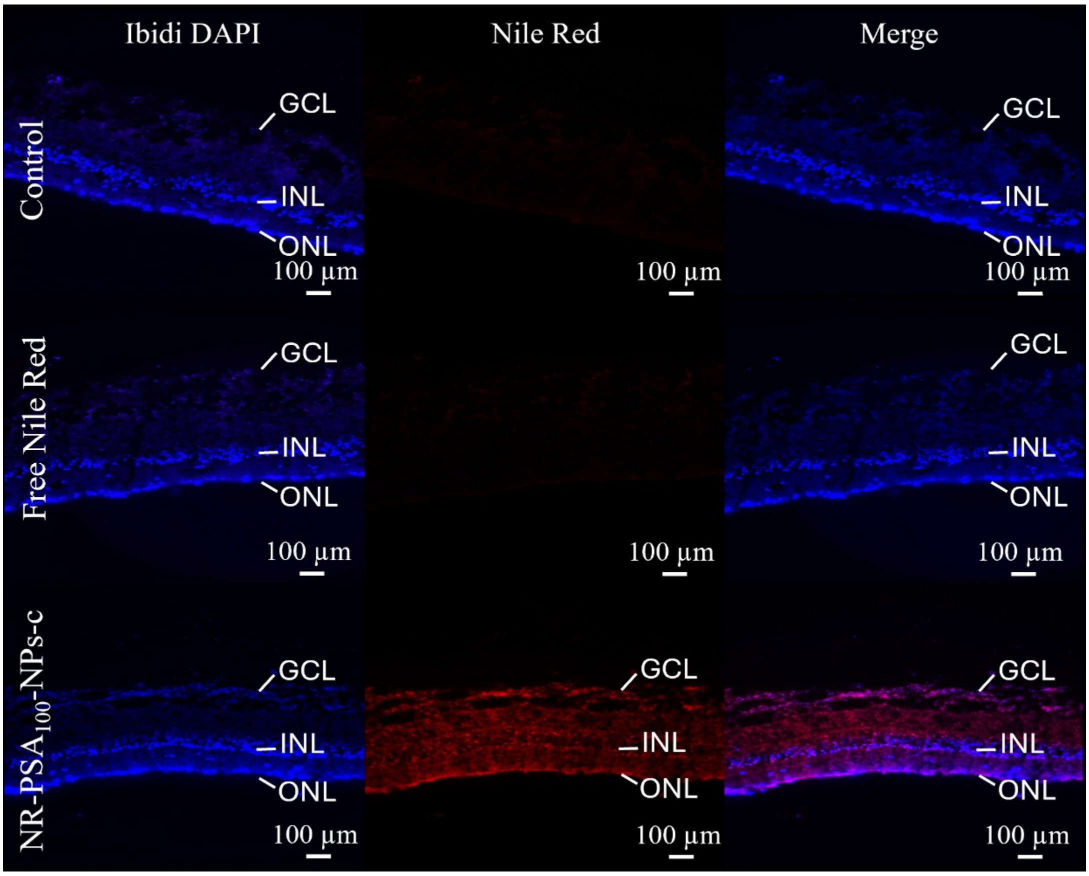

Figure S22. Porcine retina explants incubated with a) no treatment as control; b) free NR; c) NR-PSA$_{100}$-NPs-c for 24 hours. The cell nuclei of all groups were stained with DAPI. Fluorescence images (100X magnification) using blue (DAPI) and red (NR-PSA$_{100}$-NPs and free NR) channels were taken from samples, and a scale bar of 100 µm was included for reference.

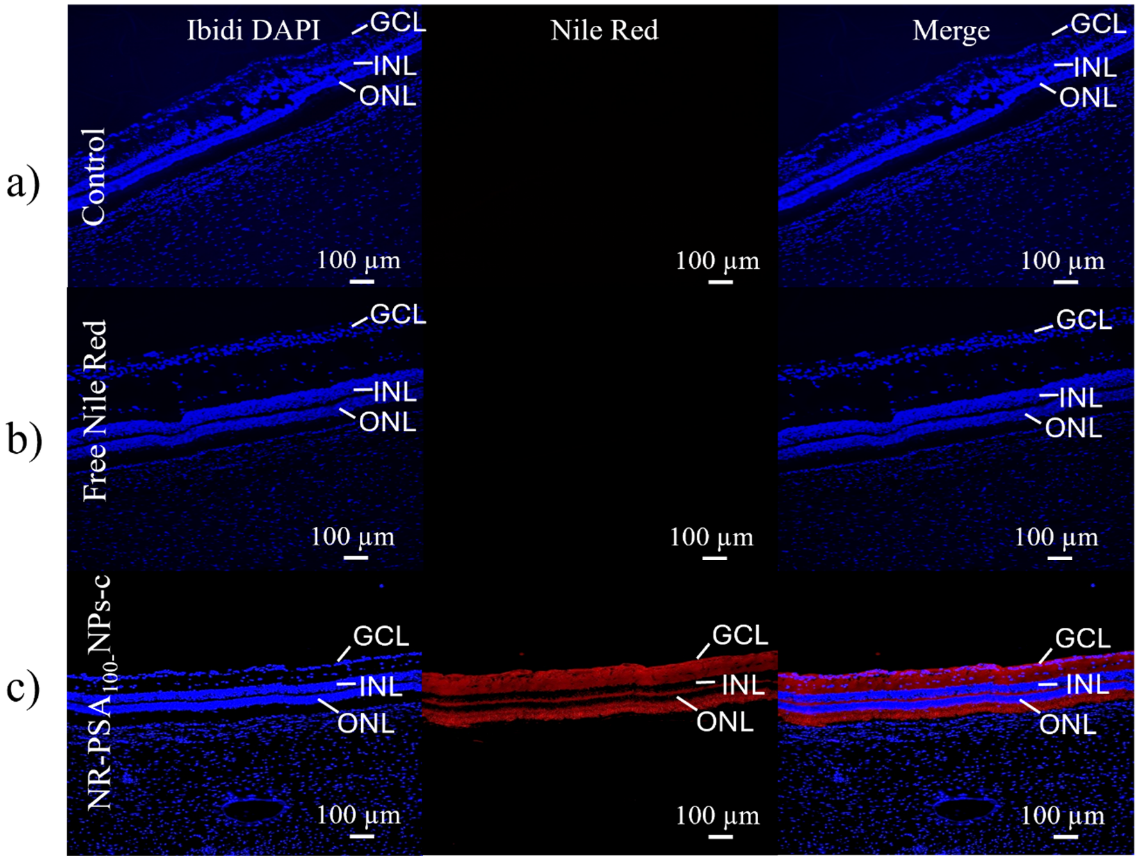

Figure S23. Dissected porcine eye without vitreous humor incubated with a) no treatment as control, b) free NR, and c) NR-PSA$_{100}$-NPs-c for 24 hours. The cell nuclei of all groups were stained with DAPI. Fluorescence images (100X magnification) using blue (DAPI) and red (NR-PSA$_{100}$-NPs and free NR) channels were taken from samples, and a scale bar of 100 μm was included for reference.

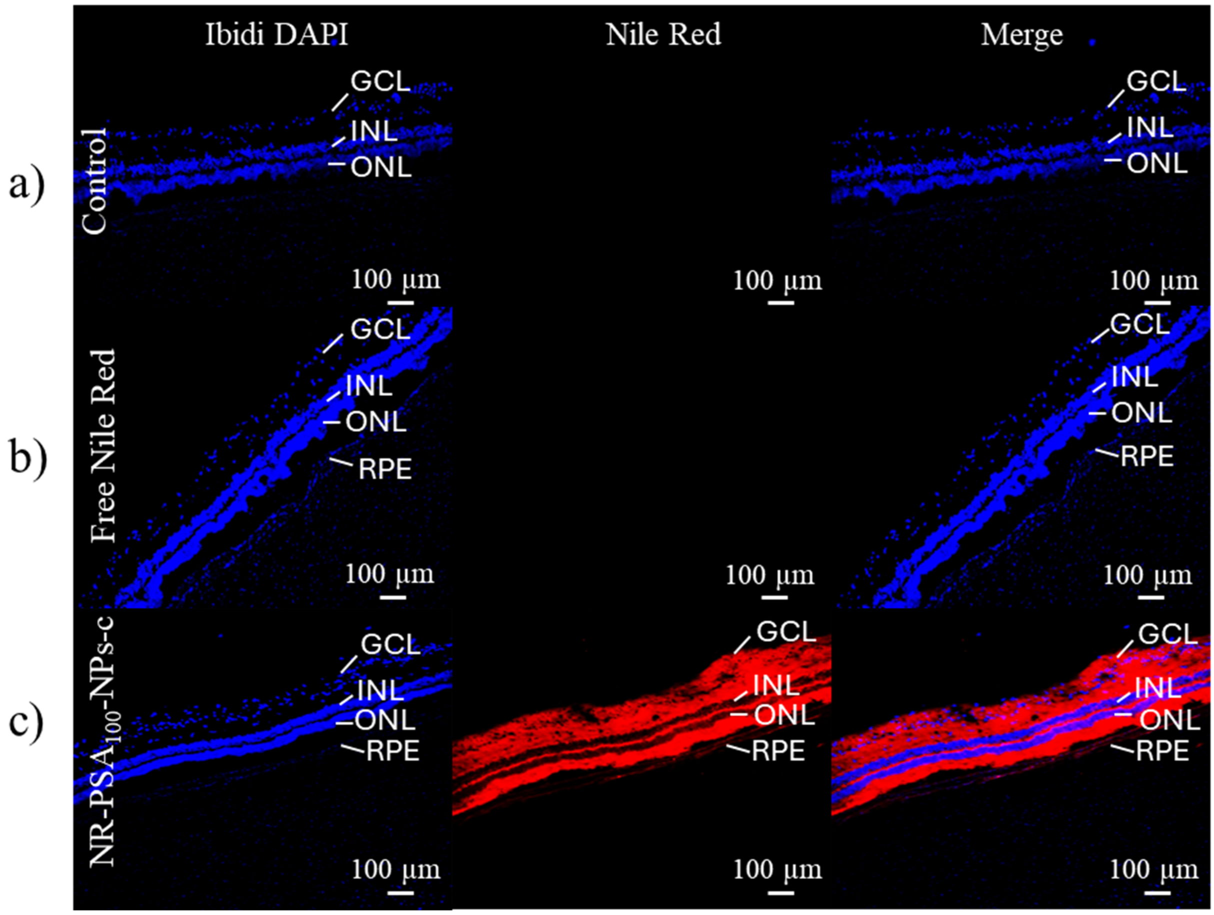

Figure S24. Complete porcine eyes were intravitreally injected with a) no treatment as control, b) free NR, and c) NR-PSA$_{100}$-NPs-c for 24 hours. The cell nuclei of all groups were stained with DAPI. Fluorescence images (100X magnification) using blue (DAPI) and red (NR-PSA$_{100}$-NPs and free NR) channels were taken from samples, and a scale bar of 100 µm was included for reference.

References

[1] A. Fietz, J. Hurst, S. Schnichels, *International Journal of Molecular Sciences* **2022**, *23*, 14540.
[2] X. Pereiro, N. Ruzafa, A. Acera, A. Urcola, E. Vecino, *Frontiers in Cellular Neuroscience* **2020**, *14*, 7.
[3] P. D. Pham, S. Monge, V. Lapinte, Y. Raoul, J. J. Robin, *European Polymer Journal* **2017**, *95*, 491.
[4] A. V. Hauck, P. Komforth, J. Erlenbusch, J. Stickdorn, K. Radacki, H. Braunschweig, P. Besenius, S. Van Herck, L. Nuhn, *Biomaterials Science* **2025**, *13*, 1414.
[5] J. Shang, X. Gao, *Chemical Society Reviews* **2014**, *43*, 7267.
[6] M. Najafi, N. Kordalivand, M.-A. Moradi, J. Van Den Dikkenberg, R. Fokkink, H. Friedrich, N. A. Sommerdijk, M. Hembury, T. Vermonden, *Biomacromolecules* **2018**, *19*, 3766.
[7] J. A. Barltrop, T. C. Owen, A. H. Cory, J. G. Cory, *Bioorganic Med. Chem. Lett.* **1991**, *1*, 611.
[8] M. Feoktistova, P. Geserick, M. Leverkus, *Cold Spring Harb Protoc* **2016**, *2016*, 343.
[9] H. J. Shin, M. Kwak, S. Joo, J. Y. Lee, *Scientific Reports* **2022**, *12*, 20146.
[10] A. Fietz, J. Hurst, S. C. Joachim, S. Schnichels, *STAR protocols* **2023**, *4*, 102443.
[11] S. Schnichels, D. Simmang, M. Löscher, A. Herrmann, J. W. de Vries, M. S. Spitzer, J. Hurst, *Pharmaceutics* **2023**, *15*, 532.
[12] V. Chourasiya, S. Bohrey, A. Pandey, *Polymers and Polymer Composites* **2021**, *29*, S1555.
[13] S. Bohrey, V. Chourasiya, A. Pandey, *Nano Converg.* **2016**, *3*, 1.
[14] P. Scholes, A. Coombes, L. Illum, S. Daviz, M. Vert, M. Davies, *Journal of controlled release* **1993**, *25*, 145.
[15] M. L. Zweers, D. W. Grijpma, G. H. Engbers, J. Feijen, *Journal of Biomedical Materials Research Part B: Applied Biomaterials: An Official Journal of The Society for Biomaterials, The Japanese Society for Biomaterials, and The Australian Society for Biomaterials and the Korean Society for Biomaterials* **2003**, *66*, 559.
[16] M. Azizi, F. Farahmandghavi, M. Joghataei, M. Zandi, M. Imani, M. Bakhtiary, F. A. Dorkoosh, F. Ghazizadeh, *J. Polym. Res.* **2013**, *20*, 1.
[17] C.-G. Keum, Y.-W. Noh, J.-S. Baek, J.-H. Lim, C.-J. Hwang, Y.-G. Na, S.-C. Shin, C.-W. Cho, *Int J Nanomedicine* **2011**, 2225.
[18] T. Wang, *Journal of Integrative Agriculture* **2019**, *18*, 1035.
[19] N. Bibi, A. ur Rehman, N. F. Rana, H. Akhtar, M. I. Khan, M. Faheem, S. B. Jamal, N. Ahmed, *Appl. Nanosci.* **2022**, *12*, 3421.
[20] A. Kyrychenko, D. A. Pasko, O. N. Kalugin, *Physical Chemistry Chemical Physics* **2017**, *19*, 8742.
[21] K. Tomoda, N. Yabuki, H. Terada, K. Makino, *Colloids and Surfaces A: Physicochemical and Engineering Aspects* **2014**, *457*, 88.
[22] E. Lepeltier, C. Bourgaux, P. Couvreur, *Advanced drug delivery reviews* **2014**, *71*, 86.
[23] B. K. Johnson, R. K. Prud'homme, *Physical review letters* **2003**, *91*, 118302.
[24] F. Bally, D. K. Garg, C. A. Serra, Y. Hoarau, N. Anton, C. Brochon, D. Parida, T. Vandamme, G. Hadziioannou, *Polymer* **2012**, *53*, 5045.
[25] R. Karnik, F. Gu, P. Basto, C. Cannizzaro, L. Dean, W. Kyei-Manu, R. Langer, O. C. Farokhzad, *Nano letters* **2008**, *8*, 2906.
[26] J. Lebouille, R. Stepanyan, J. Slot, M. C. Stuart, R. Tuinier, *Colloids and Surfaces A: Physicochemical and Engineering Aspects* **2014**, *460*, 225.
[27] W. Huang, C. Zhang, *Biotechnology journal* **2018**, *13*, 1700203.
[28] I. Takeuchi, Y. Kato, K. Makino, *Journal of oleo science* **2021**, *70*, 341.

[29] P. Shokoohinia, M. Hajialyani, K. Sadrjavadi, M. Akbari, M. Rahimi, S. Khaledian, A. Fattahi, *Research in Pharmaceutical Sciences* **2019**, *14*, 459.
[30] F. Grieser, M. Ashokkumar, J. Z. Sostaric, *Sonochemistry and sonoluminescence* **1999**, 345.
[31] N. İ. Büyük, P. Pelit, S. Derman, Z. Mustafaeva, S. Yücel, *Celal Bayar University Journal of Science* **2020**, *16*, 119.
